# Some new quantitative randomized response models using optional and partial scrambling for sensitive data

**DOI:** 10.1038/s41598-026-40714-0

**Published:** 2026-02-26

**Authors:** Shoaib Iqbal, Zawar Hussain, Talha Omer

**Affiliations:** 1 Department of Statistics, The Islamia University of Bahawalpur, Bahawalpur, 63100 Pakistan; 2 Department of Economics and Statistics, Linnaeus University, Universitetsplatsen 1, 352 52 Växjö, Sweden

**Keywords:** Computational biology and bioinformatics, Engineering, Mathematics and computing

## Abstract

This research proposes four new optional and partial quantitative randomized response models to be used for the estimation of mean and sensitivity level of quantitative variables. These models are constructed based on the current quantitative scrambling and randomization methods and seek to produce unbiased estimators with better efficiency and privacy. We compare the proposed models based on standard comparison measures, such as relative efficiency, privacy protection, and a new weighted score. The results show that proposed models provide better performance compared to the current methods and are, therefore, very appropriate for surveys dealing with sensitive information.

## Introduction and some existing quantitative models

In survey sampling, particularly when problems are sensitive in nature such as drug use, tax evasion, income, or personal behavior, direct questioning by itself is not sufficient to give reliable and honest responses. The respondents may refuse to cooperate or give dishonest answers for fear of embarrassment (see Jennings and Niemi ^1^), legal consequences, or social disapproval (see Stouffer et al. ^2^). Such non-cooperation and possible misreporting weaken the validity and accuracy of statistical conclusions drawn from the gathered data (see Hyman et al. ^3^).

To counteract such challenges, Warner ^4^ developed the Randomized Response Technique (RRT) that enabled respondents to respond to sensitive questions while maintaining their privacy and to provide an unbiased estimator of population prevalence of a sensitive attribute. Greenberg et al. ^5^ extended this methodology to the problem of estimation of mean of a sensitive quantitative variable. With the utilization of a randomizing device (such as a coin or a spinner), respondents had the ability to disguise their true answers without compromising the ability of researchers to make accurate estimates of population parameters. The RRT has come a long way since then, with several variations being added in an attempt to enhance respondent protection as well as statistical efficiency. For a detailed discussion of RRT one may refer to Fox and Tracy ^6^, Chaudhuri and Mukerjee ^7^, Lee et al. ^8^ and references cited therein.

A development that takes place in this context is the Quantitative Randomized Response Technique (QRRT), for use especially when a sensitive variable is quantitative, for example, number of criminal offenses, undeclared income, or amount of substance used. In QRRT, respondents don’t provide a straightforward numerical answer but provide a transformed value of the actual one by adding to or scrambling it with a random scrambling variable with known distribution (see Eichhorn and Hayre ^9^). This prevents individual responses from being attributed directly to the sensitive attribute and hence keeps the respondent’s privacy intact (see Nayak and Adeshiyan ^10^).

There is a disadvantage in typical initial QRRT models of using a uniform application of the scrambling technique irrespective of how comfortable or not the respondent will be in responding (see Bar-Lev et al. ^11^). Such fixity of strategy sometimes leads to lower cooperation levels and compromises on data quality. In response, researchers introduced the concept of optional modeling. The Optional Randomized Response Model (ORRM) permits respondents to decide whether or not to use the randomization mechanism, or whether to respond directly, depending on their sense of risk or discomfort (see Gupta et al. ^12^). This voluntary aspect increases respondent autonomy, diminishes psychological pressure, and may result in greater response rates and better data quality.

The Optional Quantitative Scrambling Randomized Response Models (OQSRRMs) incorporate the merits of QRRT and ORRM. These models can deal with quantitative sensitive variables while providing the respondents with an opportunity to safeguard their privacy through a scrambling mechanism (see Saha ^13^). The respondent either gives a scrambled version of his/her actual response or, if he/she feels secure, gives a direct answer. The randomization may include the addition or subtraction of a random variable, which is known by both the respondent and the researcher, or is known only by the respondent (see Gupta et al. ^14^). This randomizing factor makes it so that the individual’s actual response is indefinite, even though the value in itself is sensitive.

One of the advantages of OQSRRMs is their adaptability to any social and cultural context. In populations where the trust in confidentiality is low or stigma is prevalent, making the survey data collection optional greatly improves the rates of response (see Arnab ^15^ and Zhang et al. ^16^). Moreover, by employing an identified distribution of the scrambling variable, researchers can still achieve consistent and unbiased estimators for the total or mean of the sensitive variable in the population (see Antoch et al. ^17^). Optional scrambling thus aligns with ethical research standards and maintains scientific rigor.

Overall, OQSRRMs represent a significant leap in the evolution of privacy-preserving survey techniques. They offer a flexible, ethical, and statistically sound method for collecting truthful data on sensitive issues, enabling researchers to make informed and trustworthy conclusions about hidden aspects of society (see Heijden et al. ^18^). As survey methodology continues to evolve, these models stand out as a robust tool for addressing the dual goals of confidentiality and efficiency in statistical research (see Mulders et al. ^19^).

Over the time, RRTs have been enhanced by adding more than one stage of randomization, scrambling with correlated or unrelated variables, or mixing sensitive and non-sensitive variables. For example, Kuk ^20^ proposed a privacy-preserving technique for sensitive surveys. Christofides ^21^ created a generalized model for higher flexibility. Later research considered more complex designs. Sehra ^22^, Zahid et al. ^23^, Singh and Joarder ^24^ suggested multi-stage and mixed-question designs. Bhargava and Singh ^25^, Mangat and Singh ^26^, Upadhyaya and Singh ^27^ put their focus on efficiency and variance reduction techniques. Bar-Lev et al. ^11^ and Nayak et al. ^28^ employed statistical precision for improving estimation accuracy. Liu and Chow ^29^ covered cultural flexibility in RRMs, whereas Hussain and Shahid ^30^ implemented correlated scrambling for respondent ease. Murtaza et al. ^31^ studied the application of correlated scrambling variables to minimize bias. Singh and Sedory ^32^, Mehta et al. ^33^ proposed improved optional models for RRMs. In order to get a full understanding of the development and richness of RRMs, readers are invited to refer to Lee et al. ^8’s^ extensive review.

In this study, we propose four new quantitative randomized response models that offer unbiased estimators and improved trade-offs between privacy and efficiency. In fact, the proposed models are similar in structure, as they combine direct and scrambled responses. The scrambled responses look different but are not significantly different.

These models are designed using advanced scrambling schemes, including full and partial scrambling strategies, and are evaluated against established models. Our evaluation criteria include estimation variance, privacy measures, and a composite performance metric of Azeem ^34^, namely, the *ϕ* measure, which allows simultaneous assessment of both efficiency and privacy.

Assume the population to have *N* units and *n* units are randomly drawn from the population with replacement by simple random sampling. Let the quantitative sensitive variable of interest be denoted by *Y*, the additive and multiplicative random variables be denoted by *S* and *T*, respectively. We assume that $E\left( S \right) = 0$, $E\left( T \right) = 1$, $V\left( {Y_{i} } \right) = \sigma_{y}^{2}$, $V\left( S \right) = \sigma_{s}^{2}$, $V\left( T \right) = \sigma_{t}^{2}$, $E\left( {Y^{2} } \right) = \sigma_{y}^{2} + \mu_{y}^{2} = \mu_{2y}^{\prime }$, and $E\left( Y \right) = \mu_{y}$, where $\sigma_{y}^{2} ,\sigma_{t}^{2} ,\sigma_{s}^{2}$ are population variances of variable *Y*, *T* and *S*, respectively, and *µ*_*y*_ is the mean of the sensitive variable *Y*. Further assume that all the variables are independent of one another and some available quantitative scrambling methods exist.

## Comparison measures and some existing relevant RRMs

This section discusses the available measures of privacy and efficiency commonly used for comparing quantitative randomized response models and some of the existing RRMs.

### RRT models comparison measures

In the following subsections, we present different metrics of performance.

#### Relative efficiency

Let *MSE *(*S*) and *MSE *(*O*) be the mean squared errors of the estimators under the model of interest (Model *S*) and a reference model (Model *O*), respectively. The relative efficiency (*E*) is given by:

$$E = \frac{{MSE\left( O \right)}}{{MSE\left( S \right)}}.$$

A value *E* > 1 indicates that Model *S* is more efficient than Model *O*.

#### Yan et al. ^40^ privacy measure

To assess privacy in quantitative randomized response models, Yan et al. ^25^ proposed a metric, formally defined as:

$$\nabla = E\left( {Z - Y} \right)^{2}$$

For any randomized response model, the measure is always non-negative ($\nabla$ ≥ 0), with larger values reflecting stronger privacy protection. The metric by Yan et al. ^25^ focuses on assessing privacy.

#### Gupta et al. ^41^ combined metric

Gupta et al. ^41^ proposed a unified measure that integrates both efficiency and privacy into a single measure, defined as

$$\delta = \frac{MSE}{\nabla }.$$

The composite score δ is 0 to ∞, with smaller values indicating improved model performance in terms of reduced error, improved privacy, or both.

#### Azeem ^34^ weighted ***φ*** measure

Azeem ^19^ proposed the *ϕ*-measure, a weighted mean that balances efficiency and privacy. Let *E* be the relative efficiency, *P* the privacy ratio, and $w_{1} ,w_{2}$_*,*_ the respective weights ($w_{1} + w_{2} = 1$ =), then:

$$\phi = \frac{{w_{1} E + w_{2} P}}{{w_{1} + w_{2} }}.$$

$$\log \left( \phi \right) = \log \left( {\frac{{w_{1} E + w_{2} P}}{{w_{1} + w_{2} }}} \right)$$.

*log(ϕ)* > 0 indicates Model *S* is better than Model *O*, *log(ϕ)* = 0 implies both models perform equally, and *log(ϕ)* < 0 means Model *S* is worse than Model *O*.

#### Comparison between the combined measure δ and the weighted measure *φ*

Although the combined metric $\delta$ suggested by Gupta et al. ^41^ incorporates efficiency and privacy into a combined index, its form is rigid and does not permit adjusting the relative contribution of these two aspects. Further, the combined metric δ requires the use of mean squared error and a privacy level that do not necessarily indicate efficiency and privacy differences between competing models. In comparison, the weighted $\phi$ measure suggested by Azeem allows more flexibility for considering efficiency and privacy ratios with user-defined weights. This weighted form of $\phi$ enables researchers to draw attention to efficiency and privacy based on survey objectives. Accordingly, based on the malleability and adaptability of $\phi$ to balance efficiency and privacy, $\phi$ measure has been selected for comparison of competing models.

### Some existing RRMs

Some important and relevant RRMs have been discussed in the following subsections by giving the responses, variances of estimators and privacy measures of the models.

#### Warner ^35^ RRM

Warner ^35^ proposed a linear randomized response model that estimates sensitive population means while adding probabilistic noise to ensure unbiasedness and privacy of the respondent. The *i*^*th*^ observed response can be written as,

$$Z_{wi} = Y_{i} + S_{i} ,$$

The variance of the estimator and the corresponding privacy measure are derived to assess the efficiency and confidentiality of the method, expressed as follows:

1 $$Var\left( {\hat{\mu }_{w} } \right) = \frac{1}{n}\left( {\sigma_{y}^{2} + \sigma_{s}^{2} } \right)$$

_and_

2 $$\nabla_{w} = \sigma_{s}^{2} .$$

#### Eichhorn and Hayre ^9^ RRM

Eichhorn and Hayre ^9^ suggested a scrambled response model with multiplicative noise to conceal sensitive data while maintaining estimation quality. In this approach, the *i*^*th*^ individual’s response is randomly multiplied, and an unbiased estimator is suggested.. The *i*^*th*^ reported response under the Eichhorn and Hayre ^9^ technique is written as follows:

$$Z_{EHi} = Y_{i} T_{i} ,$$

The variance of the Eichhorn and Hayre ^9^ estimator $\hat{\mu }_{EH}$, and privacy measure are computed as:

3 $$Var\left( {\hat{\mu }_{EH} } \right) = \frac{1}{n}\left[ {\sigma_{y}^{2} + \sigma_{t}^{2} \mu_{2y}^{\prime } } \right].$$

and

4 $$\nabla_{EH} = \sigma_{t}^{2} \mu_{2y}^{\prime } .$$

#### Dian and Perri ^36^ RRM

Diana and Perri ^36^ developed a family of estimators using linear transformations of scrambled responses to estimate sensitive means while maintaining privacy. Under this model, the *i*^*th*^ respondent’s answer is generated through a linear transformation of a scrambled value, enabling unbiased estimation of the population mean. The *i*^*th*^ observed response can be written as,

$$Z_{DPi} = Y_{i} T_{i} + S_{i} ,$$

The variance of the estimator $\hat{\mu }_{DP}$ and privacy measure, $\hat{\mu }_{DP}$, of this model are given by:

5 $$Var\left( {\hat{\mu }_{DP} } \right) = \frac{1}{n}\left[ {\sigma_{y}^{2} + \sigma_{s}^{2} + \sigma_{t}^{2} \mu_{2y}^{\prime } } \right].$$

and

6 $$\nabla_{DP} = \sigma_{y}^{2} + \sigma_{s}^{2} + \sigma_{t}^{2} \mu_{2y}^{\prime } .$$

#### Gjestvang and Singh ^37^ RRM

Gjestvang and Singh ^37^ proposed a model that incorporates additive as well as multiplicative scrambling methods to enhance respondent’s privacy and accuracy in estimation. Suppose a sample of respondents is selected from the population. The respondent reports a scrambled response: with probability *P*, a positive perturbation to *Y*_*i*_*S*_*i*_ scaled by the known constant *α*, while with probability *1 − P*, a similar perturbation scaled by the known constant *β* is subtracted. Here, The *i*^*th*^ response from the respondent can be written as:

$$Z_{GSi} = \upsilon_{i} \left( {Y_{i} + \alpha Y_{i} S_{i} } \right) + \left( {1 + \upsilon_{i} } \right)\left( {Y_{i} - \beta Y_{i} S_{i} } \right),$$

where $${\upsilon }_{i}\sim Bernoulli (P)$$ distribution.

The variance of the mean estimator $$\overset{\lower0.5em\hbox{$\smash{\scriptscriptstyle\frown}$}}{\mu }_{GS}$$ and the level of privacy measure of Gjestvang and Singh ^37^ RRM are given by:

7 $$Var\left( {\overset{\lower0.5em\hbox{$\smash{\scriptscriptstyle\frown}$}}{\mu }_{GS} } \right) = \frac{1}{n}\left[ {\sigma_{y}^{2} + \alpha \beta \left( {\sigma_{s}^{2} } \right)} \right].$$

and

8 $$\nabla_{GS} = \alpha \beta \left( {\sigma_{s}^{2} } \right),$$

where α and β denote the constant and are determined by the interviewer.

#### Murtaza et al. ^38^ RRM

Murtaza et al. ^38^ presented an optimal randomized response model with correlated scrambling variables to obtain better accuracy in the estimation of sensitive means. The *i*^*th*^ response is built from a constant α and a sensitivity parameter *W*, with the possibility of balancing high privacy and low variance. Although the original model is based on correlation between scrambling variables, this study assumes independence between *T* and *S* for comparison purposes. The reported responses under the Murtaza et al. ^38^ model may be written as

$$Z_{MUi} = \nu_{i} \left( {Y_{i} } \right) + \left( {1 + \nu_{i} } \right)\left( {Y_{i} T_{i} + \alpha S_{i} } \right),$$

where $\nu_{i}$ is a Bernoulli random variable with parameter *1-W* and $\alpha$ is a constant.

The expressions for the variance of the estimator, $$\overset{\lower0.5em\hbox{$\smash{\scriptscriptstyle\frown}$}}{\mu }_{MU}$$, and the privacy measure of this model are given by:

9 $$Var\left( {\overset{\lower0.5em\hbox{$\smash{\scriptscriptstyle\frown}$}}{\mu }_{MU} } \right) = \frac{1}{n}\left[ {\sigma_{y}^{2} + W\left\{ {\alpha^{2} \sigma_{s}^{2} + \sigma_{t}^{2} \mu_{2y}^{\prime } } \right\}} \right].$$

and

10 $$\nabla_{MU} = \left\{ {\sigma_{t}^{2} \mu_{2y}^{\prime } + \alpha^{2} \sigma_{s}^{2} } \right\}.$$

#### Narjis and Shabbir ^39^ RRM

Narjis and Shabbir ^39^ also developed a scrambled randomized response model for enhancing the accuracy and confidentiality of estimating sensitive means. The model has an advantage over the previous methods in that it performs well under repeated surveys. Under this model the response of the ith respondent is generated using a multi-option randomized response mechanism to enhance privacy protection. The reported value ZNSi may take one of three forms depending on the outcome of the randomization device. The *i*^*th*^ observed response can be written as,

$$Z_{NSi} = \kappa_{1} Y_{i} + \kappa_{2} \left( {Y_{i} - \beta S_{i} } \right) + \left( {1 - \kappa_{1} - \kappa_{2} } \right)\left( {Y_{i} + \alpha S_{i} } \right),$$

where α, β and γ denote the constant and are determined by the interviewer.

The variance of the estimator $$\overset{\lower0.5em\hbox{$\smash{\scriptscriptstyle\frown}$}}{\mu }_{NS}$$ and the privacy measure of this model are given by:

11 $$Var\left( {\overset{\lower0.5em\hbox{$\smash{\scriptscriptstyle\frown}$}}{\mu }_{NS} } \right) = \frac{1}{n}\left[ {\sigma_{y}^{2} + \frac{{\alpha \beta \left( {\alpha + \beta } \right)\sigma_{s}^{2} }}{\alpha + \beta + \gamma }} \right].$$

and

12 $$\nabla_{NS} = \frac{{\alpha \beta \left( {\alpha + \beta } \right)\sigma_{s}^{2} }}{\alpha + \beta + \gamma },$$

where α, β and γ denote the constants and are determined by the interviewer.

#### Gupta et al. ^16^ RRM

The Gupta et al. ^16^ model overcomes the problem of respondent reluctance by including elements that foster trust within the survey process. It preserves the privacy of individual responses without compromising the estimation of sensitive population parameters accurately. The structure of the model provides participants with an increased sense of security, which can result in more honest answers. The model also strikes a good balance between privacy and efficiency in estimation, making it useful for real-life sensitive surveys. The Gupta et al. ^16^ Optional Enhanced Trust model addresses the issue of lack of trust by the respondent with the addition of more noise for those who do not trust Warner’s additive model. It is an optional RRT framework whereby respondents who do not perceive the question sensitive report their true value while sensitive respondents may scramble their response with one of two mechanisms depending on their trust level. Thus, the observed *i*^*th*^ response can be written as:

$$Z_{Gi} = v_{i} Y_{i} + \left( {1 - v_{i} } \right)\left[ {k_{i} \left( {Y_{i} + S_{i} } \right) + \left( {1 - k_{i} } \right)\left( {Y_{i} T_{i} + S_{i} } \right)} \right],$$

where $v_{i}$ and $k_{i}$ are Bernoulli random variables with probabilities *1-W* and *A*, respectively.

The variance of the estimator of $$\overset{\lower0.5em\hbox{$\smash{\scriptscriptstyle\frown}$}}{\mu }_{G}$$ and privacy measure of Gupta et al. ^16^ RRM are given as:

13 $$Var\left( {\overset{\lower0.5em\hbox{$\smash{\scriptscriptstyle\frown}$}}{\mu }_{G} } \right) = \frac{1}{n}\left[ {\sigma_{y}^{2} + W\sigma_{s}^{2} + W\left( {1 - A} \right)\sigma_{t}^{2} \mu_{2y}^{\prime } } \right].$$

and

14 $$\nabla_{G} = \frac{1}{n}\left[ {\sigma_{s}^{2} + \left( {1 - A} \right)\sigma_{t}^{2} \mu_{2y}^{\prime } } \right].$$

## Proposed models

In this section, four new RRMs are introduced as improvements of the methods presented by Eichhorn and Hayre ^9^, Diana and Perri ^36^, Gjestvang and Singh ^37^, and Narjis and Shabbir ^39^. The new models are designed to be more efficient than the original methods through better respondent privacy and estimation efficiency.

### Proposed model 1 (***M***_***1***_)

The Model 1 is motivated by the additive scrambling mechanism of Gjestvang and Singh ^37^. Unlike their single-stage model, the proposed Model 1 introduces a two-stage randomized response structure and modifies the response mechanism accordingly. This modification not only increases response ambiguity and enhances privacy protection but also improves estimator efficiency by better balancing the contribution of true and scrambled responses. As a result, the proposed model achieves higher efficiency while maintaining the simplicity and unbiasedness of the original Gjestvang and Singh framework.

Suppose a sample of *n* respondents is selected from the population. At the first stage, each respondent is provided with a randomization device that produces one of two statements with known probabilities $P_{1}$ and $P_{2} = 1 - P_{1}$. If the first statement, “Report the true value of the sensitive variable”, is selected, the respondent directly reports the true response *Y*_*i*_. If the second statement, “Proceed to the second randomization device”, is selected, the respondent moves to the second stage of the procedure. At the second stage, the respondent reports a scrambled response: with probability *P*, a positive perturbation to *Y*_*i*_*S*_*i*_ scaled by the known constant *α*, while with probability *1 − P*, a similar perturbation scaled by the known constant *β* is subtracted. The distribution of response from the *i*^*th*^ respondent can be written as,

$$\begin{gathered} Z_{m1i} = \left\{ {R_{1} :\left\{ {\begin{array}{*{20}c} { \, Y_{i} {\text{ with probability }}P_{1} } \\ { \, R_{2} = \left. {\left\{ {\begin{array}{*{20}c} {Y_{i} + \alpha Y_{i} S_{i} {\text{ with probability }}} \\ {Y_{i} - \beta Y_{i} S_{i} {\text{ with probability }}} \\ \end{array} \begin{array}{*{20}c} P \\ {1 - P} \\ \end{array} \, } \right.} \right\}{\text{ with probability }}P_{2} } \\ \end{array} } \right.} \right. \hfill \\ \, \hfill \\ \end{gathered}$$

The response from the *i*^*th*^ respondent may be written as:

$$Z_{m1i} = \kappa_{i} Y_{i} + \left( {1 - \kappa_{i} } \right)\left[ {\pi_{i} \left( {Y_{i} + \alpha Y_{i} S_{i} } \right) + \left( {1 - \pi_{i} } \right)\left( {Y_{i} - \beta Y_{i} S_{i} } \right)} \right],$$

where $\kappa$ and $\pi$ are Bernoulli variables with parameter $P_{1}$ and $P$, respectively.

Now, the expected response is given by

$$\begin{aligned} E\left( {Z_{m1i} } \right) = & E\left( {\kappa_{i} } \right)E\left( {Y_{i} } \right) + E\left( {1 - \kappa_{i} } \right)\left[ {E\left( {\pi_{i} } \right)\left\{ {E\left( {Y_{i} } \right) + \alpha E\left( {Y_{i} } \right)E\left( {S_{i} } \right)} \right\}} \right. \\ & \quad \quad \left. { + E\left( {1 - \pi_{i} } \right)\left\{ {E\left( {Y_{i} } \right) - \beta E\left( {Y_{i} } \right)E\left( {S_{i} } \right)} \right\}} \right] \\ \end{aligned}$$

$$E\left( {Z_{m1i} } \right) = \left( {P_{1} } \right)E\left( {Y_{i} } \right) + \left( {P_{2} } \right)\left[ {P\left( {E\left( {Y_{i} } \right)} \right) + \left( {1 - P} \right)\left( {E\left( {Y_{i} } \right)} \right)} \right],$$

$$E\left( {Z_{m1i} } \right) = E\left( {Y_{i} } \right) = \mu_{y}$$

So, by methods of moment estimation, an unbiased mean estimator of $\mu_{y}$ is given by**:**

$$\overset{\lower0.5em\hbox{$\smash{\scriptscriptstyle\frown}$}}{\mu }_{m1} = \frac{1}{n}\sum\limits_{i = 1}^{n} {Z_{m1i} } .$$.

Now, by definition, variance of the above estimator is given by

$$Var\left( {\overset{\lower0.5em\hbox{$\smash{\scriptscriptstyle\frown}$}}{\mu }_{m1} } \right) = Var\left( {\frac{1}{n}\sum\limits_{i = 1}^{n} {Z_{m1i} } } \right) = \frac{{Var\left( {Z_{m1i} } \right)}}{n}.$$

where $Var\left( {Z_{m1i} } \right)$ is given by.

$$Var\left( {Z_{m1i} } \right) = E\left( {Z^{2}_{m1i} } \right) - \left[ {E\left( {Z_{m1i} } \right)} \right]^{2}$$.

Now, consider

$$\left( {Z_{m1i}^{2} } \right) = \left[ {\kappa_{i} Y_{i} + \left( {1 - \kappa_{i} } \right)\left\{ {\pi_{i} \left( {Y_{i} + \alpha Y_{i} S_{i} } \right) + \left( {1 - \pi_{i} } \right)\left( {Y_{i} - \beta Y_{i} S_{i} } \right)} \right\}} \right]^{2} ,$$

After applying expectation,

$$E\left( {Z_{m1i}^{2} } \right) = E\left[ {\kappa_{i} Y_{i} + \left( {1 - \kappa_{i} } \right)\left\{ {\pi_{i} \left( {Y_{i} - \alpha Y_{i} S_{i} } \right) + \left( {1 - \pi_{i} } \right)\left( {Y_{i} - \beta Y_{i} S_{i} } \right)} \right\}} \right]^{2} ,$$

$$\begin{aligned} E\left( {Z_{m1i}^{2} } \right) = & E\left( {\kappa_{i}^{2} } \right)E\left( {Y_{i}^{2} } \right) + E\left( {1 - \kappa_{i} } \right)^{2} \left[ {E\left( {\pi_{i}^{2} } \right)\left\{ {E\left( {Y_{i}^{2} } \right) + \alpha^{2} E\left( {Y_{i}^{2} } \right)E\left( {S_{i}^{2} } \right)} \right\}} \right. \\ & \quad \quad \quad \left. { + E\left( {1 - \pi_{i} } \right)^{2} \left\{ {E\left( {Y_{i}^{2} } \right) + \beta^{2} E\left( {Y_{i}^{2} } \right)E\left( {S_{i}^{2} } \right)} \right\}} \right] \\ \end{aligned}$$

$$\begin{aligned} E\left( {Z_{m1i}^{2} } \right) = & \left( {P_{1} } \right)E\left( {Y_{i}^{2} } \right) + \left( {P_{2} } \right)\left[ {P\left\{ {E\left( {Y_{i}^{2} } \right) + \alpha^{2} E\left( {Y_{i}^{2} } \right)E\left( {S_{i}^{2} } \right)} \right\}} \right. \\ & \quad \quad \left. { + \left( {1 - P} \right)\left\{ {E\left( {Y_{i}^{2} } \right) + \beta^{2} E\left( {Y_{i}^{2} } \right)E\left( {S_{i}^{2} } \right)} \right\}} \right] \\ \end{aligned}$$

$$\begin{aligned} E\left( {Z_{m1i}^{2} } \right) = & \left( {P_{1} } \right)\left( {\sigma_{y}^{2} + \mu_{y}^{2} } \right) + \left( {P_{2} } \right)\left[ {\left( {\sigma_{y}^{2} + \mu_{y}^{2} } \right) + \sigma_{s}^{2} \left( {\sigma_{y}^{2} + \mu_{y}^{2} } \right)\left\{ {P\left( {\alpha^{2} - \beta^{2} } \right) + \beta^{2} } \right\}} \right] \\ { = } & \left( {\sigma_{y}^{2} + \mu_{y}^{2} } \right) + \left( {P_{2} } \right)\sigma_{s}^{2} \left( {\sigma_{y}^{2} + \mu_{y}^{2} } \right)\left\{ {P\left( {\alpha^{2} - \beta^{2} } \right) + \beta^{2} } \right\}. \\ \end{aligned}$$

Thus, the variance of ${\mathrm{Z}}_{m1i}$ is given by

$$\begin{aligned} Var\left( {Z_{m1i} } \right) = & E\left( {Z_{m1i}^{2} } \right) - \left( {E\left( {Z_{m1i} } \right)} \right)^{2} \\ { = } & \left( {\sigma_{y}^{2} + \mu_{y}^{2} } \right) + \left( {P_{2} } \right)\sigma_{s}^{2} \left( {\sigma_{y}^{2} + \mu_{y}^{2} } \right)\left\{ {P\left( {\alpha^{2} - \beta^{2} } \right) + \beta^{2} } \right\} - \mu_{y}^{2} \\ { = } & \sigma_{y}^{2} + \left( {P_{2} } \right)\sigma_{s}^{2} \left( {\sigma_{y}^{2} + \mu_{y}^{2} } \right)\left\{ {P\left( {\alpha^{2} - \beta^{2} } \right) + \beta^{2} } \right\}. \\ \end{aligned}$$

Hence, the variance of $$\overset{\lower0.5em\hbox{$\smash{\scriptscriptstyle\frown}$}}{\mu }_{m1}$$ is given by:

15 $$Var\left( {\overset{\lower0.5em\hbox{$\smash{\scriptscriptstyle\frown}$}}{\mu }_{m1} } \right) = \frac{1}{n}\left[ {\sigma_{y}^{2} + \mu_{2y}^{\prime } \left( {P_{2} \sigma_{s}^{2} } \right)\left\{ {P\left( {\alpha^{2} - \beta^{2} } \right) + \beta^{2} } \right\}} \right]$$

For the *M*_*1*_, the measure of respondent’s privacy is given by:

16 $$\nabla_{m1} = \mu_{2y}^{\prime } \left( {P_{2} \sigma_{s}^{2} } \right)\left\{ {P\left( {\alpha^{2} - \beta^{2} } \right) + \beta^{2} } \right\}$$

### Proposed model 2 (***M***_***2***_)

Model 2 is developed by taking inspiration from the model of Gupta et al. ^16^. It uses a simple probability based response system in which some respondents give the true answer, while others provide a randomized response. This structure increases privacy by hiding the true value and also helps improve efficiency by keeping part of the data unscrambled. As a result, the model builds on the idea of Gupta et al. ^16^ and offers better protection and reliability for sensitive survey questions. Each sampled respondent initially uses a randomization device that generates one of two outcomes with known probabilities $P_{1}$ and $P_{2} = 1 - P_{1}$. If direct reporting is indicated, the respondent provides the true value of the sensitive variable $Y_{i}$, otherwise, the respondent proceeds to a second randomization step. At the second stage, the response is generated through multiplicative scrambling. With probability $P$, the respondent reports $Y_{i} T_{i}$, whereas with probability $\left( {1 - P} \right)$, the respondent reports $Y_{i} \left( {1 + S_{i} } \right)$. The distribution of *i*^*th*^ response can be written as:

$$\begin{gathered} Z_{m2i} = \left\{ {R_{1} :\left\{ {\begin{array}{*{20}c} {Y_{i} {\text{ with probability }}P_{1} } \\ {R_{2} = \left\{ {\begin{array}{*{20}c} {Y_{i} T_{i} {\text{ with probability }}} \\ {Y_{i} \left( {1 + S_{i} } \right){\text{ with probability }}} \\ \end{array} \begin{array}{*{20}c} P \\ {1 - P} \\ \end{array} \, } \right.{\text{ with probability }}P_{2} } \\ \end{array} } \right.} \right. \hfill \\ \, \hfill \\ \end{gathered}$$

An unbiased mean estimator of $\mu_{y}$ under *M*_*2*_ is given by:

$$\overset{\lower0.5em\hbox{$\smash{\scriptscriptstyle\frown}$}}{\mu }_{m2} = \frac{1}{n}\sum\limits_{i = 1}^{n} {Z_{m2i} }$$

Following the steps given in subsection "Proposed model 1 (M1)", the variance of $$\overset{\lower0.5em\hbox{$\smash{\scriptscriptstyle\frown}$}}{\mu }_{m2}$$ is given by:

17 $$Var\left( {\overset{\lower0.5em\hbox{$\smash{\scriptscriptstyle\frown}$}}{\mu }_{m2} } \right) = \frac{1}{n}\left[ {\sigma_{y}^{2} + P_{2} \mu_{2y}^{\prime } \left\{ {P\sigma_{t}^{2} + \left( {1 - P} \right)\sigma_{s}^{2} } \right\}} \right]$$

For the *M*_*2*_, the measure of respondent privacy is derived as:

18 $$\nabla_{m2} = P_{2} \mu_{2y}^{\prime } \left\{ {P\sigma_{t}^{2} + \left( {1 - P} \right)\sigma_{s}^{2} } \right\}.$$

### Proposed model 3 (***M***_***3***_)

The Model 3 is developed as an extension of the Narjis and Shabbir ^39^ randomized response framework. While maintaining the original three outcome structure, the proposed model introduces an additional Bernoulli randomization device at the third outcome. Unlike the Narjis and Shabbir model, where a fixed scrambled response is reported under the third outcome, the proposed model allows two alternative responses to be selected randomly. This modification enhances response ambiguity, improves privacy protection, and provides greater flexibility in controlling the efficiency–privacy trade-off, particularly for highly sensitive survey questions.

The distribution of the *i*^*th*^ respondent’s response under the *M*_*3*_ is given as:

$$Z_{m3i} = \, \left\{ {R_{1} :\left\{ {\begin{array}{*{20}c} { Y_{i} {\text{ with probability }}P_{1} = \frac{\alpha }{\alpha + \beta + \gamma } \, } \\ { Y_{i} {\text{ + S}}_{i} {\text{ with probability }}P_{2} = \frac{\beta }{\alpha + \beta + \gamma } \, } \\ { R_{2} { = }\left\{ {\begin{array}{*{20}c} {Y_{i} T_{i} {\text{ with probability }}} \\ {Y_{i} \left( {1 + S_{i} } \right){\text{ with probability }}} \\ \end{array} \begin{array}{*{20}c} P \\ {1 - P} \\ \end{array} \, } \right.{\text{ with probability }}P_{3} = \frac{\gamma }{\alpha + \beta + \gamma } \, } \\ \end{array} } \right.} \right. \,$$

where α, β and γ are the constants and are determined by the interviewer.

The *i*^*th*^ respondent’s response may be written as

$$Z_{m3i} = \kappa_{1} Y_{i} + \kappa_{2} \left( {Y_{i} + S_{i} } \right) + \left( {1 - \kappa_{1} - \kappa_{2} } \right)\left[ {\nu_{i} \left( {Y_{i} T_{i} } \right) + \left( {1 + \nu_{i} } \right)\left( {Y_{i} + Y_{i} S_{i} } \right)} \right],$$

where $\kappa_{1}$, $\kappa_{2}$ and $\nu$ are the Bernoulli random variables with parameters $P_{1}$,$P_{2}$ and $P$, respectively.

An unbiased estimator of $\mu_{y}$ under *M*_*3*_ is given by:

$$\overset{\lower0.5em\hbox{$\smash{\scriptscriptstyle\frown}$}}{\mu }_{m3} = \frac{1}{n}\sum\limits_{i = 1}^{n} {Z_{m3i} } .$$

Again, the expression for variance of $$\overset{\lower0.5em\hbox{$\smash{\scriptscriptstyle\frown}$}}{\mu }_{m3}$$, following the steps in subsection "Proposed model 1 (M1)" is derived as:

18 $$Var\left( {\overset{\lower0.5em\hbox{$\smash{\scriptscriptstyle\frown}$}}{\mu }_{m3} } \right) = \frac{1}{n}\left[ {\sigma_{y}^{2} + P_{2} \sigma_{s}^{2} + P_{3} \mu^{\prime}_{2y} \left\{ {P\sigma_{t}^{2} + \left( {1 - P} \right)\sigma_{s}^{2} } \right\}} \right]$$

For the *M*_*3*_, the measure of respondent’s privacy is given by as:

20 $$\nabla_{m3} = P_{2} \sigma_{s}^{2} + P_{3} \mu^{\prime}_{2y} \left\{ {P\sigma_{t}^{2} + \left( {1 - P} \right)\sigma_{s}^{2} } \right\}$$

### Proposed model 4 (***M***_***4***_)

The proposed Model 4 is also motivated by the Narjis and Shabbir ^39^ randomized response framework and retains its probability-based response structure while introducing targeted enhancements at the second and third outcomes of the first randomization device. If second outcome appears, the response is modified to a multiplicative scrambling form, *Z*_*i*_ = *T*_*i*_*Y*_*i*_ ​, which effectively masks the magnitude of the sensitive variable and provides stronger privacy, particularly for large values, thereby increasing respondent confidence. In case of third outcome of the first randomization device, an additional Bernoulli randomization device is incorporated, allowing respondents to report either $Y_{i} \left( {1 + \alpha S_{i} } \right)$ or $Y_{i} \left( {1 - \beta S_{i} } \right)$, which randomizes the direction of additive scrambling and further increases response ambiguity. These modifications jointly enhance privacy protection and respondent trust while preserving the analytical simplicity and efficiency of the original Narjis and Shabbir framework.

The distribution of *i*^*th*^ respondent’s responses under the *M*_*4*_ is given as:

$$Z_{m4i} = \, \left\{ {R_{1} :\left\{ {\begin{array}{*{20}c} { Y_{i} {\text{ with probability }}P_{1} = \frac{\gamma }{\alpha + \beta + \gamma } \, } \\ { Y_{i} T_{i} {\text{ with probability }}P_{2} = \frac{\beta }{\alpha + \beta + \gamma } \, } \\ { R_{2} :\left\{ {\begin{array}{*{20}c} {Y_{i} \left( {1 + \alpha S_{i} } \right){\text{ with probability }}} \\ {Y_{i} \left( {1 - \beta S_{i} } \right){\text{ with probability }}} \\ \end{array} \begin{array}{*{20}c} P \\ {1 - P} \\ \end{array} \, } \right.{\text{with probability }}P_{3} = \frac{\alpha }{\alpha + \beta + \gamma } \, } \\ \end{array} } \right.} \right. \, {.}$$

where α, β and γ denote the constant and are determined by the interviewer.

Also, under Model 4, the *i*^*th*^ response is written as

$$Z_{m4i} = \kappa_{1} Y_{i} + \kappa_{2} \left( {Y_{i} T_{i} } \right) + \left( {1 - \kappa_{1} - \kappa_{2} } \right)\left[ {\nu \left( {Y_{i} + \alpha Y_{i} S_{i} } \right) + \left( {1 + \nu } \right)\left( {Y_{i} - \beta Y_{i} S_{i} } \right)} \right],$$

where $\kappa_{1}$, $\kappa_{2}$ and $\nu$ are defined as in Model 3.

An unbiased estimator of $\mu_{y}$ under *M*_*4*_ is given as:

$$\overset{\lower0.5em\hbox{$\smash{\scriptscriptstyle\frown}$}}{\mu }_{m4} = \frac{1}{n}\sum\limits_{i = 1}^{n} {Z_{m4i} } .$$

As earlier, following the steps in subsection "Proposed model 1 (M1)", the algebraic expression for variance of $$\overset{\lower0.5em\hbox{$\smash{\scriptscriptstyle\frown}$}}{\mu }_{m4}$$ is derived as

21 $$Var\left( {\overset{\lower0.5em\hbox{$\smash{\scriptscriptstyle\frown}$}}{\mu }_{m4} } \right) = \frac{1}{n}\left[ {\sigma_{y}^{2} + P_{2} \left( {\sigma_{y}^{2} + \mu_{y}^{2} } \right)\sigma_{t}^{2} + \left( {P_{3} \sigma_{s}^{2} \mu_{2y}^{\prime } } \right)\left\{ {P\left( {\alpha^{2} - \beta^{2} } \right) + \beta^{2} } \right\}} \right]$$

For the *M*_*4*_, the measure of respondent’s privacy is given by :

22 $$\nabla_{m4} = \left[ {P_{2} \left( {\sigma_{y}^{2} + \mu_{y}^{2} } \right)\sigma_{t}^{2} + \left( {P_{3} \sigma_{s}^{2} \mu_{2y}^{\prime } } \right)\left\{ {P\left( {\alpha^{2} - \beta^{2} } \right) + \beta^{2} } \right\}} \right]$$

## Comparison of models using the Phi ($\phi$) measure

Section Comparison of models using the Phi(ϕ) measure presents a detailed comparative performance analysis of the proposed randomized response models. For clarity of presentation, each proposed model is examined separately against the same set of seven existing randomized response models. Accordingly, Sections "Comparison of considered existing models with " to "Comparison of considered existing models with The comparison of the proposed model 4 with the existing models considered in this study using the measure is given below" are devoted to the comparative analyses of Models *M*_*1*_*, M*_*2*_*, M*_*3*_*,* and *M*_*4*_, respectively. Within each section, the corresponding sub-sections provide one-to-one comparisons between the proposed model and each existing model under identical design parameter settings. The comparisons are carried out using the combined performance measure $\log \left( \phi \right)$, allowing a consistent assessment of efficiency and privacy characteristics across all models. This structured approach ensures transparency and facilitates straightforward interpretation of the comparative results.

### Comparison of considered existing models with $M_{1}$

In this subsection, we give the comparison of existing models with the proposed Model 1.

#### The $\phi$ measure of warner ^35^ model relative to the M_1_

The efficiency and privacy of the Warner ^35^ model relative to the *M*_*1*_ using (1), (2), (15) and (16) can be expressed as:

23 $$E_{{\left( {W/M_{1} } \right)}} = \frac{{\sigma_{y}^{2} + \sigma_{s}^{2} }}{{\sigma_{y}^{2} + \mu_{2y}^{\prime } \left( {P_{2} \sigma_{s}^{2} } \right)\left\{ {P\left( {\alpha^{2} - \beta^{2} } \right) + \beta^{2} } \right\}}}$$

and

24 $$P_{{\left( {W/M_{1} } \right)}} = \frac{{\mu_{2y}^{\prime } \left( {P_{2} \sigma_{s}^{2} } \right)\left\{ {P\left( {\alpha^{2} - \beta^{2} } \right) + \beta^{2} } \right\}}}{{\sigma_{s}^{2} }}$$

Using (23) and (24), the mathematical expression for *ϕ* can be written as:

25 $$\phi_{{\left( {W/M_{1} } \right)}} = \frac{1}{{\left( {w_{1} + w_{2} } \right)}}\left( {w_{1} E_{{\left( {W/M_{1} } \right)}} + w_{2} P_{{\left( {W/M_{1} } \right)}} } \right)$$

#### The $\phi$ measure of Eichhorn and Hayre ^9^ model relative to the ***M***_***1***_

The efficiency and privacy of the Eichhorn and Hayre ^9^ model relative to the *M*_*1*_ using (3), (4), (15) and (16) can be expressed as:

26 $$E_{{\left( {EH/M_{1} } \right)}} = \frac{{\sigma_{y}^{2} + \sigma_{t}^{2} \mu_{2y}^{\prime } }}{{\sigma_{y}^{2} + \mu_{2y}^{\prime } \left( {P_{2} \sigma_{s}^{2} } \right)\left\{ {P\left( {\alpha^{2} - \beta^{2} } \right) + \beta^{2} } \right\}}}$$

and

27 $$P_{{\left( {EH/M_{1} } \right)}} = \frac{{\left( {P_{2} \sigma_{s}^{2} } \right)\left\{ {P\left( {\alpha^{2} - \beta^{2} } \right) + \beta^{2} } \right\}}}{{\sigma_{t}^{2} }}$$

Now, using (26) and (27), the mathematical expression for *ϕ* can be written as:

28 $$\phi_{{\left( {EH/M_{1} } \right)}} = \frac{1}{{\left( {w_{1} + w_{2} } \right)}}\left( {w_{1} E_{{\left( {EH/M_{1} } \right)}} + w_{2} P_{{\left( {EH/M_{1} } \right)}} } \right)$$

#### The $\phi$ Measure of Diana and Perri ^36^ model relative to the ***M***_***1***_

The efficiency and privacy of the Diana and Perri ^36^ model relative to the *M*_*1*_ using (5), (6), (15) and (16) can be expressed as:

29 $$E_{{\left( {DP/M_{1} } \right)}} = \frac{{\sigma_{y}^{2} + \sigma_{s}^{2} + \sigma_{t}^{2} \mu_{2y}^{\prime } }}{{\sigma_{y}^{2} + \mu_{2y}^{\prime } \left( {P_{2} \sigma_{s}^{2} } \right)\left\{ {P\left( {\alpha^{2} - \beta^{2} } \right) + \beta^{2} } \right\}}}$$

and

30 $$P_{{\left( {DP/M_{1} } \right)}} = \frac{{\mu_{2y}^{\prime } \left( {P_{2} \sigma_{s}^{2} } \right)\left\{ {P\left( {\alpha^{2} - \beta^{2} } \right) + \beta^{2} } \right\}}}{{\sigma_{s}^{2} + \sigma_{t}^{2} \mu_{2y}^{\prime } }}{.}$$

Using (29) and (30), the mathematical expression for *ϕ* can be written as:

31 $$\phi_{{\left( {DP/M_{1} } \right)}} = \frac{1}{{\left( {w_{1} + w_{2} } \right)}}\left( {w_{1} E_{{\left( {DP/M_{1} } \right)}} + w_{2} P_{{\left( {DP/M_{1} } \right)}} } \right)$$

#### The $\phi$ Measure of Gjestvang and Singh ^37^ Model relative to the ***M***_***1***_

The efficiency and privacy of the Gjestvang and Singh ^37^ model relative to the *M*_*1*_ using (7), (8), (15) and (16) can be expressed as:

32 $$E_{{\left( {GS/M_{1} } \right)}} = \frac{{\sigma_{y}^{2} + \alpha \beta \left( {\sigma_{s}^{2} } \right)}}{{\sigma_{y}^{2} + \mu_{2y}^{\prime } \left( {P_{2} \sigma_{s}^{2} } \right)\left\{ {P\left( {\alpha^{2} - \beta^{2} } \right) + \beta^{2} } \right\}}}$$

and

33 $$P_{{\left( {GS/M_{1} } \right)}} = \frac{{\mu_{2y}^{\prime } \left( {P_{2} \sigma_{s}^{2} } \right)\left\{ {P\left( {\alpha^{2} - \beta^{2} } \right) + \beta^{2} } \right\}}}{{\alpha \beta \left( {\sigma_{s}^{2} } \right)}}$$

Using (32) and (33), the mathematical expression for *ϕ* can be written as:

34 $$\phi_{{\left( {GS/M_{1} } \right)}} = \frac{1}{{\left( {w_{1} + w_{2} } \right)}}\left( {w_{1} E_{{\left( {GS/M_{1} } \right)}} + w_{2} P_{{\left( {GS/M_{1} } \right)}} } \right)$$

#### The $\phi$ measure of Murtaza et al. ^38^ model relative to the ***M***_***1***_

The efficiency and privacy of the Murtaza et al. ^38^ model relative to the *M*_*1*_ using (9), (10), (15) and (16) can be expressed as:

35 $$E_{{\left( {MU/M_{1} } \right)}} = \frac{{\sigma_{y}^{2} + W\left\{ {\alpha^{2} \sigma_{s}^{2} + \sigma_{t}^{2} \mu_{2y}^{\prime } } \right\}}}{{\sigma_{y}^{2} + \mu_{2y}^{\prime } \left( {P_{2} \sigma_{s}^{2} } \right)\left\{ {P\left( {\alpha^{2} - \beta^{2} } \right) + \beta^{2} } \right\}}}$$

and

36 $$P_{{\left( {MU/M_{1} } \right)}} = \frac{{\mu_{2y}^{\prime } \left( {P_{2} \sigma_{s}^{2} } \right)\left\{ {P\left( {\alpha^{2} - \beta^{2} } \right) + \beta^{2} } \right\}}}{{\left\{ {\alpha^{2} \sigma_{s}^{2} + \sigma_{t}^{2} \mu_{2y}^{\prime } } \right\}}}{.}$$

Using (35) and (36), the mathematical expression for *ϕ* can be written as:

37 $$\phi_{{\left( {MU/M_{1} } \right)}} = \frac{1}{{\left( {w_{1} + w_{2} } \right)}}\left( {w_{1} E_{{\left( {MU/M_{1} } \right)}} + w_{2} P_{{\left( {MU/M_{1} } \right)}} } \right)$$

#### The $\phi$ measure of Narjis and Shabbir ^39^ model relative to the ***M***_***1***_

The efficiency and privacy of the Narjis and Shabbir ^39^ model relative to the *M*_1_ using (11), (12), (15) and (16) can be expressed as:

38 $$E_{{\left( {NS/M_{1} } \right)}} = \frac{{\left[ {\left( {\alpha + \beta + \gamma } \right)\sigma_{y}^{2} + \alpha \beta \left( {\alpha + \beta } \right)\sigma_{s}^{2} } \right]}}{{\left( {\alpha + \beta + \gamma } \right)\left[ {\sigma_{y}^{2} + \mu_{2y}^{\prime } \left( {P_{2} \sigma_{s}^{2} } \right)\left\{ {P\left( {\alpha^{2} - \beta^{2} } \right) + \beta^{2} } \right\}} \right]}}$$

and

39 $$P_{{\left( {NS/M_{1} } \right)}} = \frac{{\left( {\alpha + \beta + \gamma } \right)\mu_{2y}^{\prime } \left( {P_{2} \sigma_{s}^{2} } \right)\left\{ {P\left( {\alpha^{2} - \beta^{2} } \right) + \beta^{2} } \right\}}}{{\alpha \beta \left( {\alpha + \beta } \right)\sigma_{s}^{2} }}$$

Using (38) and (39), the mathematical expression for *ϕ* can be written as:

40 $$\phi_{{\left( {NS/M_{1} } \right)}} = \frac{1}{{\left( {w_{1} + w_{2} } \right)}}\left( {w_{1} E_{{\left( {NS/M_{1} } \right)}} + w_{2} P_{{\left( {NS/M_{1} } \right)}} } \right)$$

#### The Phi measure of Gupta et al. ^16^ model relative to the ***M***_***1***_

The efficiency and privacy of the Gupta et al. ^16^ model relative to the *M*_*1*_ using (13), (14), (15) and (16) can be expressed as:

41 $$E_{{\left( {G/M_{1} } \right)}} = \frac{{\left[ {\sigma_{y}^{2} + W\sigma_{s}^{2} + W\left( {1 - A} \right)\sigma_{t}^{2} \mu_{2y}^{\prime } } \right]}}{{\sigma_{y}^{2} + \mu_{2y}^{\prime } \left( {P_{2} \sigma_{s}^{2} } \right)\left\{ {P\left( {\alpha^{2} - \beta^{2} } \right) + \beta^{2} } \right\}}}$$

and

42 $$P_{{\left( {G/M_{1} } \right)}} = \frac{{\mu_{2y}^{\prime } \left( {P_{2} \sigma_{s}^{2} } \right)\left\{ {P\left( {\alpha^{2} - \beta^{2} } \right) + \beta^{2} } \right\}}}{{\left[ {\sigma_{s}^{2} + \left( {1 - A} \right)\sigma_{t}^{2} \mu_{2y}^{\prime } } \right]}}$$

Using (41) and (42), the mathematical expression for *ϕ* can be written as:

43 $$\phi_{{\left( {G/M_{1} } \right)}} = \frac{1}{{\left( {w_{1} + w_{2} } \right)}}\left( {w_{1} E_{{\left( {G/M_{1} } \right)}} + w_{2} P_{{\left( {G/M_{1} } \right)}} } \right)$$

**Fig. 1:**
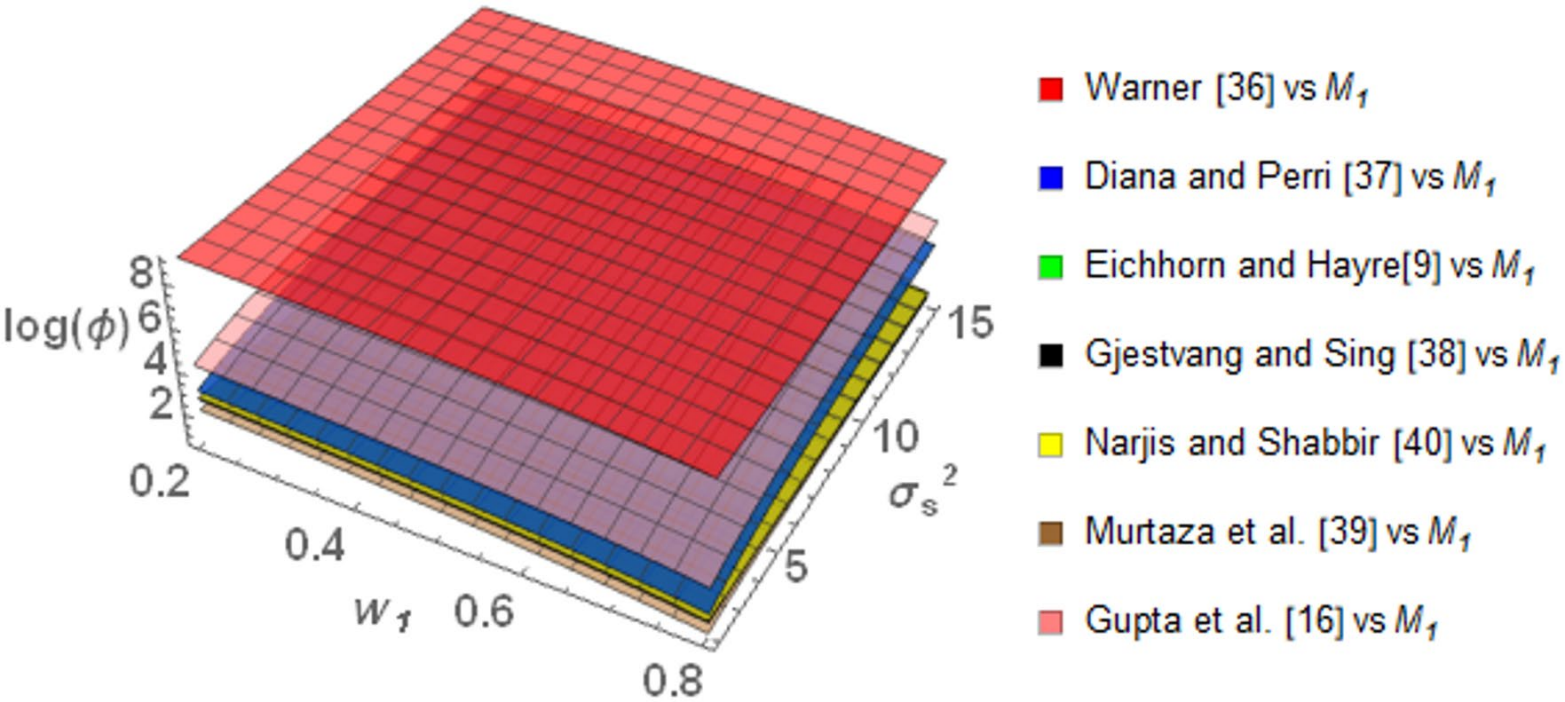
Behavior of $\log \left( \phi \right)$ for Model *M*_*1*_ relative to the existing models for $\sigma_{y}^{2} = 2$, $\mu_{y} = 5$, $\sigma_{T}^{2} = 25$, $\alpha = 25$, $\beta = 25$, $\gamma = 5$, $P = 0.4$, $P_{1} = 0.4$, $P_{2} = 0.6$, $A = 0.7$ A = 0.7, and $W = 0.2.$.

The Fig. 1 portrays the behavior of $\log \left( \phi \right)$ for the proposed model *M*_*1*_ relative to the existing models discussed above. This comparison uses the $\log \left( \phi \right)$ value to evaluate both efficiency and privacy. A positive $\log \left( \phi \right)$ value indicates that *M*_*1*_ outperforms the others in terms of these criteria, whereas a negative value suggests inferior performance. The graphs demonstrate that *M*_*1*_ generally performs better than the majority of the compared models, as indicated by consistently positive *log(ϕ)* values. Specifically, when compared with Warner ^35^ model, *M*_*1*_ shows superior performance at lower values of the weighting parameter $w_{1}$, where $\log \left( \phi \right)$ reaches approximately 3. Although $\log \left( \phi \right)$ gradually decreases as $w_{1}$ increases, it remains positive throughout the range of $w_{1}$, signifying continued better relative performance. Similar trends are observed when comparing *M*_*1*_ with those by Eichhorn and Hayre^9^, Diana and Perri^36^, and Murtaza et al. ^38^. In these instances, $\log \left( \phi \right)$ remains positive with the increase in $w_{1}$, showing steady performance. Further, the $\log \left( \phi \right)$ value is seen to decrease as the $\sigma_{S}^{2}$ increases but $\log \left( \phi \right)$ remain positive, supporting the strength of *M*_*1*_ in different conditions. *M*_*1*_ also shows better performance compared to Narjis and Shabbir ^39^ and Gjestvang and Singh ^37^ models. The Fig. 1 shows that as $w_{1}$ increase, the $\log \left( \phi \right)$ for *M*_*1*_ also increases, reaching a value of approximately 2.5 when $w_{1}$ is 0.8. It performs better than Gupta et al. ^16^ model when $w_{1}$ ranges between 0.65 and 0.8 and $\sigma_{S}^{2}$ is below 2.4. Outside of this range, when $w_{1}$ is less than 0.65 or when $\sigma_{S}^{2}$ is above 2.4, the Gupta et al. ^16^ model performs better.

The model *M*_*1*_ exhibits overall better performance with respect to efficiency as well as privacy compared to a number of well-known randomized response models. Its consistently positive values of $\log \left( \phi \right)$ across different combinations of the design parameters indicate the stability of the model.

### Comparison of considered existing models with $M_{2}$

Comparison of existing models with the proposed Model 2 is given in the following sub-sections.

#### The $\phi$ measure of warner ^35^ model relative to the ***M***_***2***_

The efficiency and privacy of the Warner ^35^ model relative to the *M*_*2*_ using (1), (2), (17) and (18), can be expressed as:

44 $$E_{{\left( {W/M_{2} } \right)}} = \frac{{\sigma_{y}^{2} + \sigma_{s}^{2} }}{{\sigma_{y}^{2} + P_{2} \mu_{2y}^{\prime } \left\{ {P\sigma_{t}^{2} + \left( {1 - P} \right)\sigma_{s}^{2} } \right\}}}$$

and

45 $$P_{{\left( {W/M_{2} } \right)}} = \frac{{P_{2} \mu_{2y}^{\prime } \left\{ {P\sigma_{t}^{2} + \left( {1 - P} \right)\sigma_{s}^{2} } \right\}}}{{\sigma_{s}^{2} }}$$

Using the Eq. (44) and (45), the mathematical expression for *ϕ* can be written as:

46 $$\phi_{{\left( {W/M_{2} } \right)}} = \frac{1}{{\left( {w_{1} + w_{2} } \right)}}\left( {w_{1} E_{{\left( {W/M_{2} } \right)}} + w_{2} P_{{\left( {W/M_{2} } \right)}} } \right)$$

#### The $\phi$ measure of Eichhorn and Hayre ^9^ model relative to the ***M***_***2***_

The efficiency and privacy of the Eichhorn and Hayre ^9^ model relative to the *M*_*2*_ using (3), (4), (17) and (18), can be expressed as:

47 $$E_{{\left( {EH/M_{2} } \right)}} = \frac{{\sigma_{y}^{2} + \sigma_{t}^{2} \mu_{2y}^{\prime } }}{{\sigma_{y}^{2} + P_{2} \mu_{2y}^{\prime } \left\{ {P\sigma_{t}^{2} + \left( {1 - P} \right)\sigma_{s}^{2} } \right\}}}$$

and

48 $$P_{{\left( {EH/M_{2} } \right)}} = \frac{{P_{2} \left\{ {P\sigma_{t}^{2} + \left( {1 - P} \right)\sigma_{s}^{2} } \right\}}}{{\sigma_{t}^{2} }}$$

Using (47) and (48), the mathematical expression for *ϕ* can be written as:

49 $$\phi_{{\left( {EH/M_{2} } \right)}} = \frac{1}{{\left( {w_{1} + w_{2} } \right)}}\left( {w_{1} E_{{\left( {EH/M_{2} } \right)}} + w_{2} P_{{\left( {EH/M_{2} } \right)}} } \right){ (49)}$$

#### The $\phi$ measure of Diana and Perri ^36^ model relative to the ***M***_***2***_

The efficiency and privacy of the Diana and Perri ^36^ model relative to the *M*_*2*_ using (5), (6), (17) and (18) can be expressed as:

50 $$E_{{\left( {DP/M_{2} } \right)}} = \frac{{\sigma_{y}^{2} + \sigma_{s}^{2} + \sigma_{t}^{2} \mu_{2y}^{\prime } }}{{\sigma_{y}^{2} + P_{2} \mu_{2y}^{\prime } \left\{ {P\sigma_{t}^{2} + \left( {1 - P} \right)\sigma_{s}^{2} } \right\}}}$$

and

51 $$P_{{\left( {DP/M_{2} } \right)}} = \frac{{P_{2} \mu_{2y}^{\prime } \left\{ {P\sigma_{t}^{2} + \left( {1 - P} \right)\sigma_{s}^{2} } \right\}}}{{\sigma_{s}^{2} + \sigma_{t}^{2} \mu_{2y}^{\prime } }}$$

Using the Eq. (50) and (51), the mathematical expression for *ϕ* can be written as:

52 $$\phi_{{\left( {DP/M_{2} } \right)}} = \frac{1}{{\left( {w_{1} + w_{2} } \right)}}\left( {w_{1} E_{{\left( {DP/M_{2} } \right)}} + w_{2} P_{{\left( {DP/M_{2} } \right)}} } \right)$$

#### The $\phi$ Measure of Gjestvang and Singh^37^ Model Relative to the ***M***_***2***_

The efficiency and privacy of the Gjestvang and Singh^37^ model relative to the *M*_*2*_ using (7), (8), (17) and (18), can be expressed as:

53 $$E_{{\left( {GS/M_{2} } \right)}} = \frac{{\sigma_{y}^{2} + \alpha \beta \left( {\sigma_{s}^{2} } \right)}}{{\sigma_{y}^{2} + P_{2} \mu_{2y}^{\prime } \left\{ {P\sigma_{t}^{2} + \left( {1 - P} \right)\sigma_{s}^{2} } \right\}}}$$

and

54 $$P_{{\left( {GS/M_{2} } \right)}} = \frac{{P_{2} \mu_{2y}^{\prime } \left\{ {p\sigma_{t}^{2} + \left( {1 - p} \right)\sigma_{s}^{2} } \right\}}}{{\alpha \beta \left( {\sigma_{s}^{2} } \right)}}$$

Using (53) and (54), the mathematical expression for *ϕ* can be written as:

55 $$\phi_{{\left( {GS/M_{2} } \right)}} = \frac{1}{{\left( {w_{1} + w_{2} } \right)}}\left( {w_{1} E_{{\left( {GS/M_{2} } \right)}} + w_{2} P_{{\left( {GS/M_{2} } \right)}} } \right)$$

**4.2.5. The** $\phi$ **Measure of Murtaza et al. **^38^** Model relative to the M**_***2***_***.***

The efficiency and privacy of the Murtaza et al. ^38^ model relative to the *M*_*2*_ using (9), (10), (17) and (18), can be expressed as:

56 $$E_{{\left( {MU/M_{2} } \right)}} = \frac{{\sigma_{y}^{2} + W\left\{ {\alpha^{2} \sigma_{s}^{2} + \sigma_{t}^{2} \mu_{2y}^{\prime } } \right\}}}{{\sigma_{y}^{2} + P_{2} \mu_{2y}^{\prime } \left\{ {P\sigma_{t}^{2} + \left( {1 - P} \right)\sigma_{s}^{2} } \right\}}}$$

and

57 $$P_{{\left( {MU/M_{2} } \right)}} = \frac{{P_{2} \mu_{2y}^{\prime } \left\{ {P\sigma_{t}^{2} + \left( {1 - P} \right)\sigma_{s}^{2} } \right\}}}{{\left\{ {\alpha^{2} \sigma_{s}^{2} + \sigma_{t}^{2} \mu_{2y}^{\prime } } \right\}}}$$

Using (56) and (57), the mathematical expression for *ϕ* can be written as:

58 $$\phi_{{\left( {MU/M_{2} } \right)}} = \frac{1}{{\left( {w_{1} + w_{2} } \right)}}\left( {w_{1} E_{{\left( {MU/M_{2} } \right)}} + w_{2} P_{{\left( {MU/M_{2} } \right)}} } \right)$$

#### The $\phi$ Measure of Narjis and Shabbir ^39^ Model relative to the ***M***_***2***_

The efficiency and privacy of the Narjis and Shabbir ^39^ model relative to the *M*_*2*_ using (11), (12), (17) and (18), can be expressed as:

59 $$E_{{\left( {NS/M_{2} } \right)}} = \frac{{\left[ {\left( {\alpha + \beta + \gamma } \right)\sigma_{y}^{2} + \alpha \beta \left( {\alpha + \beta } \right)\sigma_{s}^{2} } \right]}}{{\left( {\alpha + \beta + \gamma } \right)\left[ {\sigma_{y}^{2} + P_{2} \mu_{2y}^{\prime } \left\{ {P\sigma_{t}^{2} + \left( {1 - P} \right)\sigma_{s}^{2} } \right\}} \right]}}$$

and

60 $$P_{{\left( {NS/M_{2} } \right)}} = \frac{{\left( {\alpha + \beta + \gamma } \right)P_{2} \mu_{2y}^{\prime } \left\{ {P\sigma_{t}^{2} + \left( {1 - P} \right)\sigma_{s}^{2} } \right\}}}{{\alpha \beta \left( {\alpha + \beta } \right)\sigma_{s}^{2} }}$$

Using (59) and (60), the mathematical expression for *ϕ* can be written as:

61 $$\phi_{{\left( {NS/M_{2} } \right)}} = \frac{1}{{\left( {w_{1} + w_{2} } \right)}}\left( {w_{1} E_{{\left( {NS/M_{2} } \right)}} + w_{2} P_{{\left( {NS/M_{2} } \right)}} } \right)$$

#### The $\phi$ Measure of Gupta et al. ^16^ Model relative to the ***M***_***2***_

The efficiency and privacy of the Gupta et al. ^16^ model relative to the proposed *M*_*2*_ using (13), (14), (17) and (18), can be expressed as:

62 $$E_{{\left( {G/M_{2} } \right)}} = \frac{{\left[ {\sigma_{y}^{2} + W\sigma_{s}^{2} + W\left( {1 - A} \right)\sigma_{t}^{2} \mu_{2y}^{\prime } } \right]}}{{\sigma_{y}^{2} + P_{2} \mu_{2y}^{\prime } \left\{ {P\sigma_{t}^{2} + \left( {1 - P} \right)\sigma_{s}^{2} } \right\}}}$$

and

63 $$P_{{\left( {G/M_{2} } \right)}} = \frac{{P_{2} \mu_{2y}^{\prime } \left\{ {P\sigma_{t}^{2} + \left( {1 - P} \right)\sigma_{s}^{2} } \right\}}}{{\left[ {\sigma_{s}^{2} + \left( {1 - A} \right)\sigma_{t}^{2} \mu_{2y}^{\prime } } \right]}}$$

Using (62) and (63), the mathematical expression for *ϕ* can be written as:

64 $$\phi_{{\left( {G/M_{2} } \right)}} = \frac{1}{{\left( {w_{1} + w_{2} } \right)}}\left( {w_{1} E_{{\left( {G/M_{2} } \right)}} + w_{2} P_{{\left( {G/M_{2} } \right)}} } \right)$$

**Fig. 2:**
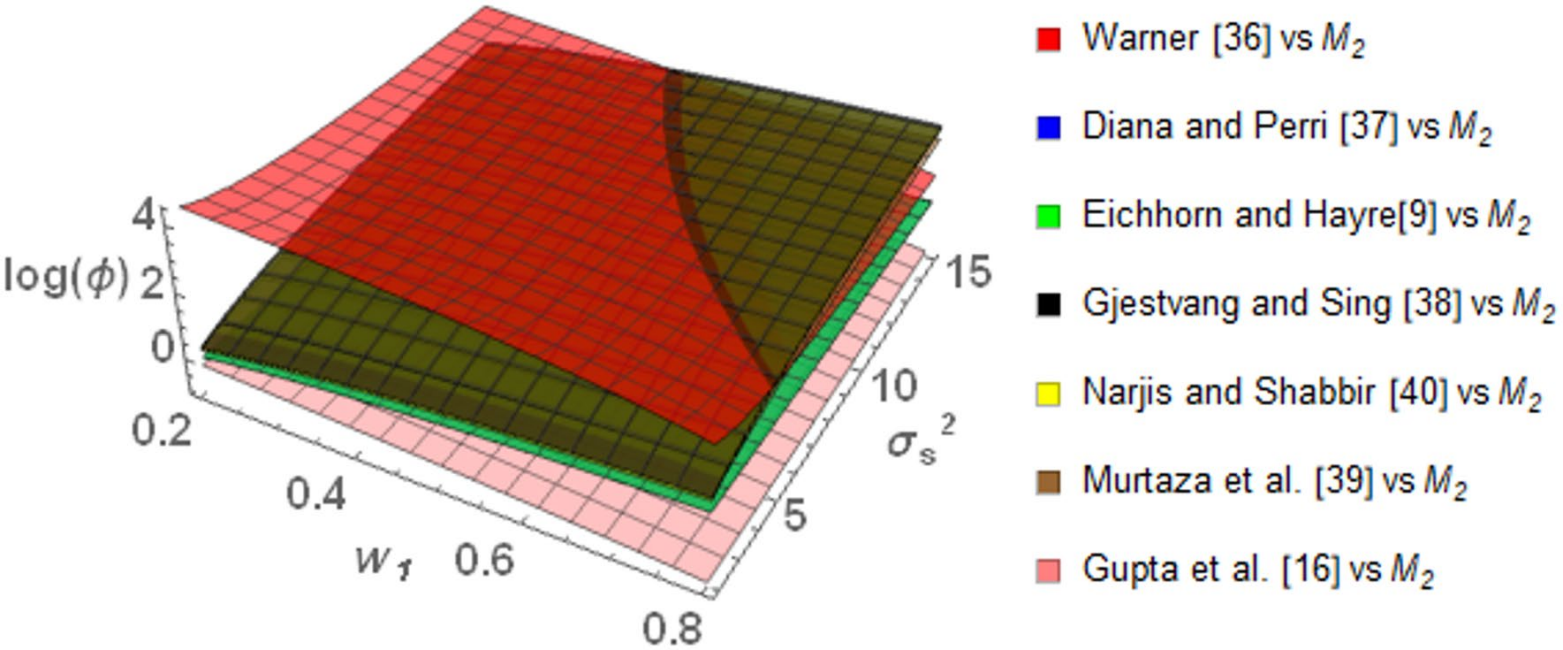
Behavior of $\log \left( \phi \right)$ for Model *M*_*2*_ relative to the existing models for $\sigma_{y}^{2} = 2$, $\mu_{y} = 5$, $\sigma_{T}^{2} = 25$, $\alpha = 25$, $\beta = 25$, $\gamma = 5$, $P = 0.4$, $P_{1} = 0.4$, $P_{2} = 0.6$, $A = 0.7$ A = 0.7, and $W = 0.2.$.

Figure 2 illustrates the behavior of $\log \left( \phi \right)$ for the proposed model *M*_*2*_ in comparison with the existing models mentioned earlier. Specifically, compared to Warner ^35^, *M*_*2*_ is highly efficient at lower values of the weighting parameter $w_{1}$, with $\log \left( \phi \right)$ maximizing around 4. Even as $w_{1}$ and $\sigma_{S}^{2}$ increase, $\log \left( \phi \right)$ decreases but still remains positive , which suggests consistent efficiency.

Compared to Eichhorn and Hayre ^9^, Diana and Perri ^36^, Gjestvang and Singh ^37^, Narjis and Shabbir ^39^ and Murtaza et al. ^38^, *M*_*2*_ performs better throughout the range of $w_{1}$ and $\sigma_{S}^{2}$. So as $w_{1}$ increases the value of $\log \left( \phi \right)$ increases which suggest that *M*_*2*_ performs well. The value of $\log \left( \phi \right)$ of *M*_*2*_ as compared to Eichhorn and Hayre ^9^, Diana and Perri ^36^ decreases but remains positive as $\sigma_{S}^{2}$ increases but compared to Gjestvang and Singh ^37^, Narjis and Shabbir ^39^ and Murtaza et al. ^38^, *M*_*2*_ shows better performance when $\sigma_{S}^{2}$ increases. Finally, as compared to Gupta et al. ^16^, *M*_*2*_ performs better if $w_{1}$ remains less than 0.27 and $\sigma_{S}^{2}$ remains greater than 10. Otherwise, Gupta et al. ^16^ is more efficient as compare to *M*_*2.*_

### Comparison of considered existing models with $M_{3}$

Comparison of existing models with the proposed Model 3 is given in the following sub-sub-sections.

#### The $\phi$ Measure of Warner ^35^ Model relative to the ***M***_***3***_

The efficiency and privacy of the Warner ^35^ model relative to the *M*_*3*_ using (1), (2), (19) and (20) can be expressed as:

65 $$E_{{\left( {W/M_{3} } \right)}} = \frac{{\sigma_{y}^{2} + \sigma_{s}^{2} }}{{\sigma_{y}^{2} + P_{2} \sigma_{s}^{2} + P_{3} \mu_{2y}^{\prime } \left\{ {P\sigma_{t}^{2} + \left( {1 - P} \right)\sigma_{s}^{2} } \right\}}}$$

and

66 $$P_{{\left( {W/M_{3} } \right)}} = \frac{{P_{2} \sigma_{s}^{2} + P_{3} \mu_{2y}^{\prime } \left\{ {P\sigma_{t}^{2} + \left( {1 - P} \right)\sigma_{s}^{2} } \right\}}}{{\sigma_{s}^{2} }}$$

Using (65) and (66), the mathematical expression for *ϕ* can be written as:

67 $$\phi_{{\left( {W/M_{3} } \right)}} = \frac{1}{{\left( {w_{1} + w_{2} } \right)}}\left( {w_{1} E_{{\left( {W/M_{3} } \right)}} + w_{2} P_{{\left( {W/M_{3} } \right)}} } \right)$$

#### The $\phi$ Measure of Eichhorn and Hayre ^9^ Model Relative to the ***M***_***3***_

The efficiency and privacy of the Eichhorn and Hayre ^9^ model relative to the *M*_*3*_ using (3), (4), (19) and (20) can be expressed as:

68 $$E_{{\left( {EH/M_{3} } \right)}} = \frac{{\sigma_{y}^{2} + \sigma_{t}^{2} \mu_{2y}^{\prime } }}{{\sigma_{y}^{2} + P_{2} \sigma_{s}^{2} + P_{3} \mu_{2y}^{\prime } \left\{ {P\sigma_{t}^{2} + \left( {1 - P} \right)\sigma_{s}^{2} } \right\}}}$$

and

69 $$P_{{\left( {EH/M_{3} } \right)}} = \frac{{P_{2} \sigma_{s}^{2} + P_{3} \mu_{2y}^{\prime } \left\{ {P\sigma_{t}^{2} + \left( {1 - P} \right)\sigma_{s}^{2} } \right\}}}{{\sigma_{t}^{2} \mu_{2y}^{\prime } }}$$

Using (68) and (69), the mathematical expression for *ϕ* can be written as:

70 $$\phi_{{\left( {EH/M_{3} } \right)}} = \frac{1}{{\left( {w_{1} + w_{2} } \right)}}\left( {w_{1} E_{{\left( {EH/M_{3} } \right)}} + w_{2} P_{{\left( {EH/M_{3} } \right)}} } \right)$$

#### The $\phi$ Measure of Diana and Perri ^36^ Model relative to the ***M***_***3***_

The efficiency and privacy of the Diana and Perri ^36^ model relative to the *M*_*3*_ using (5), (6), (19) and (20) can be expressed as:

71 $$E_{{\left( {DP/M_{3} } \right)}} = \frac{{\sigma_{y}^{2} + \sigma_{s}^{2} + \sigma_{t}^{2} \mu_{2y}^{\prime } }}{{\left\{ {\sigma_{y}^{2} + P_{2} \sigma_{s}^{2} + P_{3} \mu_{2y}^{\prime } \left\{ {P\sigma_{t}^{2} + \left( {1 - P} \right)\sigma_{s}^{2} } \right\}} \right\}}}$$

and

72 $$P_{{\left( {DP/M_{3} } \right)}} = \frac{{P_{2} \sigma_{s}^{2} + P_{3} \mu_{2y}^{\prime } \left\{ {P\sigma_{t}^{2} + \left( {1 - P} \right)\sigma_{s}^{2} } \right\}}}{{\sigma_{s}^{2} + \sigma_{t}^{2} \mu_{2y}^{\prime } }}$$

Using (71) and (72), the mathematical expression for *ϕ* can be written as:

73 $$\phi_{{\left( {DP/M_{3} } \right)}} = \frac{1}{{\left( {w_{1} + w_{2} } \right)}}\left( {w_{1} E_{{\left( {DP/M_{3} } \right)}} + w_{2} P_{{\left( {DP/M_{3} } \right)}} } \right)$$

#### The $\phi$ Measure of Gjestvang and Singh ^37^ Model relative to the ***M***_***3***_

The efficiency and privacy of the Gjestvang and Singh ^37^ model relative to the *M*_*3*_ using (7), (8), (19) and (20) can be expressed as:

74 $$E_{{\left( {GS/M_{3} } \right)}} = \frac{{\sigma_{y}^{2} + \alpha \beta \left( {\sigma_{s}^{2} } \right)}}{{\sigma_{y}^{2} + P_{2} \sigma_{s}^{2} + P_{3} \mu_{2y}^{\prime } \left\{ {P\sigma_{t}^{2} + \left( {1 - P} \right)\sigma_{s}^{2} } \right\}}}$$

and

75 $$P_{{\left( {GS/M_{3} } \right)}} = \frac{{P_{2} \sigma_{s}^{2} + P_{3} \mu_{2y}^{\prime } \left\{ {P\sigma_{t}^{2} + \left( {1 - P} \right)\sigma_{s}^{2} } \right\}}}{{\alpha \beta \left( {\sigma_{s}^{2} } \right)}}$$

Using (74) and (75), the mathematical expression for *ϕ* can be written as:

76 $$\phi_{{\left( {GS/M_{3} } \right)}} = \frac{1}{{\left( {w_{1} + w_{2} } \right)}}\left( {w_{1} E_{{\left( {GS/M_{3} } \right)}} + w_{2} P_{{\left( {GS/M_{3} } \right)}} } \right)$$

#### The $\phi$ Measure of Murtaza et al. ^38^ Model relative to the ***M***_***3***_

The efficiency and privacy of the Murtaza et al. ^38^ model relative to the *M*_*3*_ using (9), (10), (19) and (20) can be expressed as:

77 $$E_{{\left( {MU/M_{3} } \right)}} = \frac{{\sigma_{y}^{2} + P_{2} \left\{ {\alpha^{2} \sigma_{s}^{2} + \sigma_{t}^{2} \mu_{2y}^{\prime } } \right\}}}{{\sigma_{y}^{2} + P_{2} \sigma_{s}^{2} + P_{3} \mu_{2y}^{\prime } \left\{ {P\sigma_{t}^{2} + \left( {1 - P} \right)\sigma_{s}^{2} } \right\}}}$$

and

78 $$P_{{\left( {MU/M_{3} } \right)}} = \frac{{P_{2} \sigma_{s}^{2} + P_{3} \mu_{2y}^{\prime } \left\{ {P\sigma_{t}^{2} + \left( {1 - P} \right)\sigma_{s}^{2} } \right\}}}{{P_{2} \left\{ {\alpha^{2} \sigma_{s}^{2} + \sigma_{t}^{2} \mu_{2y}^{\prime } } \right\}}}$$

Using (77) and (78), the mathematical expression for *ϕ* can be written as:

79 $$\phi_{{\left( {MU/M_{3} } \right)}} = \frac{1}{{\left( {w_{1} + w_{2} } \right)}}\left( {w_{1} E_{{\left( {MU/M_{3} } \right)}} + w_{2} P_{{\left( {MU/M_{3} } \right)}} } \right)$$

#### The $\phi$ Measure of Narjis and Shabbir ^39^ Model relative to the ***M***_***3***_

The efficiency and privacy of the Narjis and Shabbir ^39^ model relative to the *M*_*8*_ using (11), (12), (19) and (20) can be expressed as:

80 $$E_{{\left( {NS/M_{3} } \right)}} = \frac{{\left[ {\left( {\alpha + \beta + \gamma } \right)\sigma_{y}^{2} + \alpha \beta \left( {\alpha + \beta } \right)\sigma_{s}^{2} } \right]}}{{\left( {\alpha + \beta + \gamma } \right)\left[ {\sigma_{y}^{2} + P_{2} \sigma_{s}^{2} + P_{3} \mu_{2y}^{\prime } \left\{ {P\sigma_{t}^{2} + \left( {1 - P} \right)\sigma_{s}^{2} } \right\}} \right]}}$$

and

81 $$P_{{\left( {NS/M_{3} } \right)}} = \frac{{\left( {\alpha + \beta + \gamma } \right)\left[ {P_{2} \sigma_{s}^{2} + P_{3} \mu_{2y}^{\prime } \left\{ {P\sigma_{t}^{2} + \left( {1 - P} \right)\sigma_{s}^{2} } \right\}} \right]}}{{\alpha \beta \left( {\alpha + \beta } \right)\sigma_{s}^{2} }}$$

Using (80) and (81), the mathematical expression for *ϕ* can be written as:

82 $$\phi_{{\left( {NS/M_{3} } \right)}} = \frac{1}{{\left( {w_{1} + w_{2} } \right)}}\left( {w_{1} E_{{\left( {NS/M_{3} } \right)}} + w_{2} P_{{\left( {NS/M_{3} } \right)}} } \right)$$

#### The $\phi$ Measure of Gupta et al. ^16^ Model relative to the ***M***_***3***_

The efficiency and privacy of the Gupta et al. ^16^ model relative to the *M*_*3*_ using (13), (14), (19) and (20) can be expressed as:

83 $$E_{{\left( {G/M_{3} } \right)}} = \frac{{\left[ {\sigma_{y}^{2} + W\sigma_{s}^{2} + W\left( {1 - A} \right)\sigma_{t}^{2} \mu_{2y}^{\prime } } \right]}}{{\sigma_{y}^{2} + P_{2} \sigma_{s}^{2} + P_{3} \mu_{2y}^{\prime } \left\{ {P\sigma_{t}^{2} + \left( {1 - P} \right)\sigma_{s}^{2} } \right\}}}$$

and

84 $$P_{{\left( {G/M_{3} } \right)}} = \frac{{P_{2} \sigma_{s}^{2} + P_{3} \mu_{2y}^{\prime } \left\{ {P\sigma_{t}^{2} + \left( {1 - P} \right)\sigma_{s}^{2} } \right\}}}{{\left[ {\sigma_{s}^{2} + \left( {1 - A} \right)\sigma_{t}^{2} \mu_{2y}^{\prime } } \right]}}$$

Using (83) and (84), the mathematical expression for *ϕ* can be written as:

85 $$\phi_{{\left( {G/M_{3} } \right)}} = \frac{1}{{\left( {w_{1} + w_{2} } \right)}}\left( {w_{1} E_{{\left( {G/M_{3} } \right)}} + w_{2} P_{{\left( {G/M_{3} } \right)}} } \right)$$

**Fig. 3:**
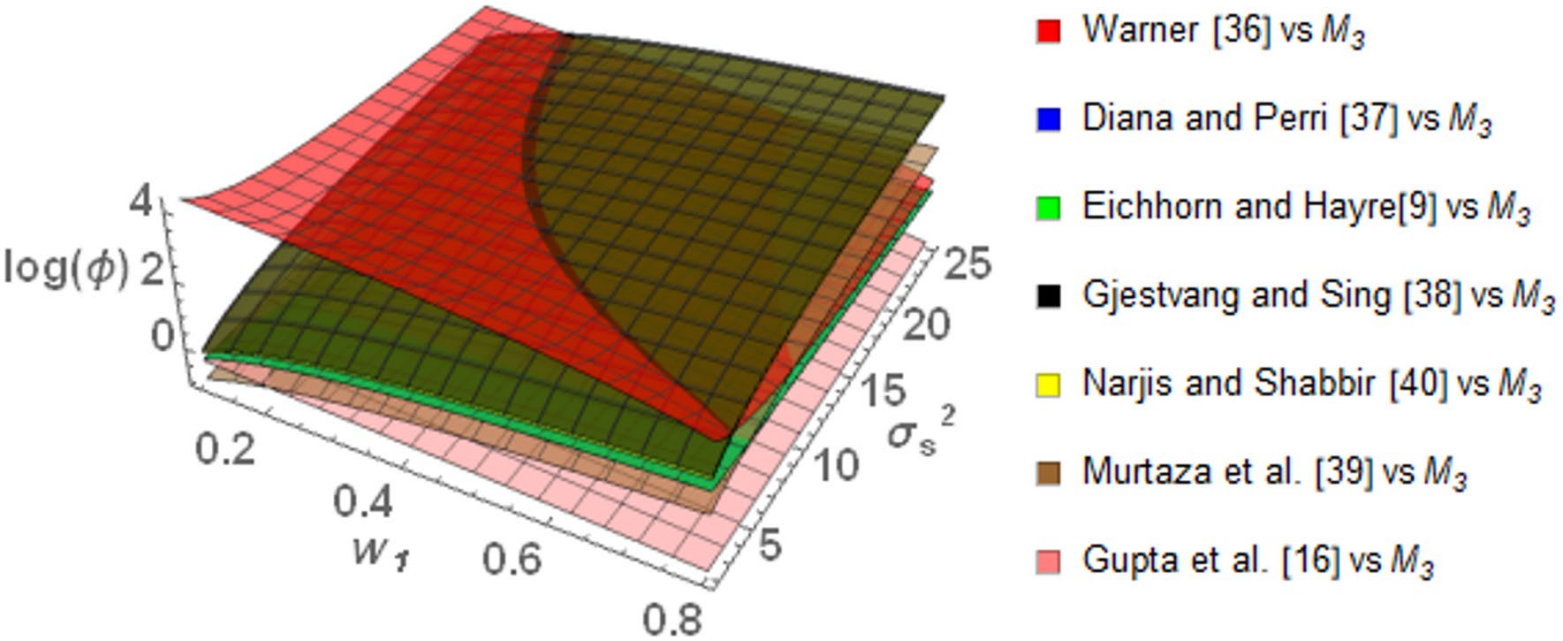
Behavior of $\log \left( \phi \right)$ for Model *M*_*3*_ relative to the existing models for $\sigma_{y}^{2} = 2$, $\mu_{y} = 5$, $\sigma_{T}^{2} = 25$, $\alpha = 25$, $\beta = 25$, $\gamma = 5$, $P = 0.4$, $P_{1} = 0.4$, $P_{2} = 0.6$, $A = 0.7$ A = 0.7, and $W = 0.2.$.

The graphs in the Fig. 3 show that *M*_*3*_ always has relatively better performance compared to the other models considered in this study, as evident from the mainly positive *log(ϕ)* values. Especially, when compared with Warner ^35^, *M*_*3*_ is most efficient at lower $w_{1}$ with $\log \left( \phi \right)$ up to about 4. While $\log \left( \phi \right)$ slightly decreases as $w_{1}$ and $\sigma_{S}^{2}$ increases, it remains above zero, indicating that* M*_*3*_ maintains its advantage across the spectrum. *M*_*3*_ performed better than Eichhorn and Hayre ^9^ and Diana and Perri ^36^ throughout the range of $w_{1}$ and $\sigma_{S}^{2}$*. M*_*3*_ performs more effectively when $w_{1}$ increases and $\sigma_{S}^{2}$ decreases. The *M*_*3*_ also performs better relative to Gjestvang and Singh ^37^ and Narjis and Shabbir ^39^ models across a wide range of different parametric values. Specifically, for $w_{1}$ between 0.2 and 0.8 and $\sigma_{S}^{2}$ ranging from 5 to 20, *M*_*3*_ maintains a positive $\log \left( \phi \right)$. On the higher extremes of $w_{1}$, such as $w_{1} = 0.8$, $\log \left( \phi \right)$ tends towards 3, emphasizing the good performance of *M*_*3*_ under different conditions. In comparison to Murtaza et al. ^38^, *M*_*3*_ is less efficient when $w_{1}$ is less than 0.25 since $\log \left( \phi \right)$ falls below zero. Nevertheless, *M*_*3*_ outperforms this model when $w_{1}$ is greater than 0.25. In comparison to Gupta et al. ^16^, *M*_*3*_ performs well for $w_{1}$ less than 0.2 while keeping $\log \left( \phi \right)$ positive. Over this threshold, $\log \left( \phi \right)$ becomes negative, which indicates a relative decrease in the performance of *M*_*3*_.

### Comparison of considered existing models with $M_{4}$ The comparison of the proposed model 4 with the existing models considered in this study using the $\phi$ measure is given below

#### The $\phi$ Measure of Warner ^35^ Model relative to the ***M***_***4***_

The efficiency and privacy of the Warner ^35^ model relative to the *M*_*4*_ using (1), (2), (21) and (22) can be expressed as:

86 $$E_{{\left( {W/M_{4} } \right)}} = \frac{{\sigma_{y}^{2} + \sigma_{s}^{2} }}{{\sigma_{y}^{2} + P_{2} \mu_{2y}^{\prime } \sigma_{t}^{2} + \left( {P_{3} \sigma_{s}^{2} } \right)\mu_{2y}^{\prime } \left\{ {P\left( {\alpha^{2} - \beta^{2} } \right) + \beta^{2} } \right\}}}$$

and

87 $$P_{{\left( {W/M_{4} } \right)}} = \frac{{P_{2} \mu_{2y}^{\prime } \sigma_{t}^{2} + \left( {P_{3} \sigma_{s}^{2} } \right)\mu_{2y}^{\prime } \left\{ {P\left( {\alpha^{2} - \beta^{2} } \right) + \beta^{2} } \right\}}}{{\sigma_{s}^{2} }}$$

Using (86) and (87), the mathematical expression for *ϕ* can be written as:

88 $$\phi_{{\left( {W/M_{4} } \right)}} = \frac{1}{{\left( {w_{1} + w_{2} } \right)}}\left( {w_{1} E_{{\left( {W/M_{4} } \right)}} + w_{2} P_{{\left( {W/M_{4} } \right)}} } \right)$$

#### The $\phi$ Measure of Eichhorn and Hayre ^9^ Model relative to the ***M***_***4***_

The efficiency and privacy of the Eichhorn and Hayre ^9^ model relative to the *M*_*4*_ using (3), (4), (21) and (22) can be expressed as:

89 $$E_{{\left( {EH/M_{4} } \right)}} = \frac{{\sigma_{y}^{2} + \sigma_{t}^{2} \mu_{2y}^{\prime } }}{{\sigma_{y}^{2} + P_{2} \mu_{2y}^{\prime } \sigma_{t}^{2} + \left( {P_{3} \sigma_{s}^{2} } \right)\mu_{2y}^{\prime } \left\{ {P\left( {\alpha^{2} - \beta^{2} } \right) + \beta^{2} } \right\}}}$$

and

90 $$P_{{\left( {EH/M_{4} } \right)}} = \frac{{P_{2} \mu_{2y}^{\prime } \sigma_{t}^{2} + \left( {P_{3} \sigma_{s}^{2} } \right)\mu_{2y}^{\prime } \left\{ {P\left( {\alpha^{2} - \beta^{2} } \right) + \beta^{2} } \right\}}}{{\sigma_{t}^{2} \mu_{2y}^{\prime } }}$$

Using (89) and (90), the mathematical expression for *ϕ* can be written as:

91 $$\phi_{{\left( {EH/M_{4} } \right)}} = \frac{1}{{\left( {w_{1} + w_{2} } \right)}}\left( {w_{1} E_{{\left( {EH/M_{4} } \right)}} + w_{2} P_{{\left( {EH/M_{4} } \right)}} } \right)$$

#### The $\phi$ Measure of Diana and Perri ^36^ Model relative to the ***M***_***4***_

The efficiency and privacy of the Diana and Perri ^36^ model relative to the *M*_*9*_ using (5), (6), (21) and (22) can be expressed as:

92 $$E_{{\left( {DP/M_{4} } \right)}} = \frac{{\sigma_{y}^{2} + \sigma_{s}^{2} + \sigma_{t}^{2} \mu_{2y}^{\prime } }}{{\sigma_{y}^{2} + P_{2} \mu_{2y}^{\prime } \sigma_{t}^{2} + \left( {P_{3} \sigma_{s}^{2} } \right)\mu^{\prime}_{2y} \left\{ {P\left( {\alpha^{2} - \beta^{2} } \right) + \beta^{2} } \right\}}}$$

and

93 $$P_{{\left( {DP/M_{4} } \right)}} = \frac{{P_{2} \mu_{2y}^{\prime } \sigma_{t}^{2} + \left( {P_{3} \sigma_{s}^{2} } \right)\mu_{2y}^{\prime } \left\{ {P\left( {\alpha^{2} - \beta^{2} } \right) + \beta^{2} } \right\}}}{{\sigma_{s}^{2} + \sigma_{t}^{2} \mu_{2y}^{\prime } }}$$

Using (92) and (93), the mathematical expression for *ϕ* can be written as:

94 $$\phi_{{\left( {DP/M_{4} } \right)}} = \frac{1}{{\left( {w_{1} + w_{2} } \right)}}\left( {w_{1} E_{{\left( {DP/M_{4} } \right)}} + w_{2} P_{{\left( {DP/M_{4} } \right)}} } \right)$$

#### The $\phi$ Measure of Gjestvang and Singh ^37^ Model relative to the ***M***_***4***_

The efficiency and privacy of the Gjestvang and Singh ^37^ model relative to the *M*_*4*_ using (7), (8), (21) and (22) can be expressed as:

95 $$E_{{\left( {GS/M_{4} } \right)}} = \frac{{\sigma_{y}^{2} + \alpha \beta \left( {\sigma_{s}^{2} } \right)}}{{\sigma_{y}^{2} + P_{2} \mu_{2y}^{\prime } \sigma_{t}^{2} + \left( {P_{3} \sigma_{s}^{2} } \right)\mu_{2y}^{\prime } \left\{ {P\left( {\alpha^{2} - \beta^{2} } \right) + \beta^{2} } \right\}}}$$

and

96 $$P_{{\left( {GS/M_{4} } \right)}} = \frac{{\sigma_{y}^{2} + P_{2} \mu^{\prime}_{2y} \sigma_{t}^{2} + \left( {P_{3} \sigma_{s}^{2} } \right)\mu_{2y}^{\prime } \left\{ {P\left( {\alpha^{2} - \beta^{2} } \right) + \beta^{2} } \right\}}}{{\alpha \beta \left( {\sigma_{s}^{2} } \right)}}$$

Using the Eqs. (95) and (96), the mathematical expression for *ϕ* can be written as:

97 $$\phi_{{\left( {GS/M_{4} } \right)}} = \frac{1}{{\left( {w_{1} + w_{2} } \right)}}\left( {w_{1} E_{{\left( {GS/M_{4} } \right)}} + w_{2} P_{{\left( {GS/M_{4} } \right)}} } \right)$$

#### The $\phi$ Measure of Murtaza et al. ^38^ Model relative to the ***M***_***4***_

The efficiency and privacy of the Murtaza et al. ^38^ model relative to the *M*_*4*_ using (9), (10), (21) and (22) can be expressed as:

98 $$E_{{\left( {MU/M_{4} } \right)}} = \frac{{\sigma_{y}^{2} + W\left\{ {\alpha^{2} \sigma_{s}^{2} + \sigma_{t}^{2} \mu_{2y}^{\prime } } \right\}}}{{\sigma_{y}^{2} + P_{2} \mu_{2y}^{\prime } \sigma_{t}^{2} + \left( {P_{3} \sigma_{s}^{2} } \right)\mu_{2y}^{\prime } \left\{ {P\left( {\alpha^{2} - \beta^{2} } \right) + \beta^{2} } \right\}}}$$

and

99 $$P_{{\left( {MU/M_{4} } \right)}} = \frac{{P_{2} \mu_{2y}^{\prime } \sigma_{t}^{2} + \left( {P_{3} \sigma_{s}^{2} } \right)\mu_{2y}^{\prime } \left\{ {P\left( {\alpha^{2} - \beta^{2} } \right) + \beta^{2} } \right\}}}{{\left\{ {\alpha^{2} \sigma_{s}^{2} + \sigma_{t}^{2} \mu_{2y}^{\prime } } \right\}}}$$

Using (98) and (99), the mathematical expression for *ϕ* can be written as:

100 $$\phi_{{\left( {MU/M_{4} } \right)}} = \frac{1}{{\left( {w_{1} + w_{2} } \right)}}\left( {w_{1} E_{{\left( {MU/M_{4} } \right)}} + w_{2} P_{{\left( {MU/M_{4} } \right)}} } \right)$$

#### The $\phi$ Measure of Narjis and Shabbir ^39^ Model relative to the ***M***_***4***_

The efficiency and privacy of the Narjis and Shabbir ^39^ model relative to the *M*_*4*_ using (11), (12), (21) and (22) can be expressed as:

101 $$E_{{\left( {NS/M_{4} } \right)}} = \frac{{\left[ {\left( {\alpha + \beta + \gamma } \right)\sigma_{y}^{2} + \alpha \beta \left( {\alpha + \beta } \right)\sigma_{s}^{2} } \right]}}{{\left( {\alpha + \beta + \gamma } \right)\left[ {\sigma_{y}^{2} + P_{2} \mu_{2y}^{\prime } \sigma_{t}^{2} + \left( {P_{3} \sigma_{s}^{2} } \right)\mu_{2y}^{\prime } \left\{ {P\left( {\alpha^{2} - \beta^{2} } \right) + \beta^{2} } \right\}} \right]}}$$

and

102 $$P_{{\left( {NS/M_{4} } \right)}} = \frac{{\left( {\alpha + \beta + \gamma } \right)\left[ {P_{2} \mu_{2y}^{\prime } \sigma_{t}^{2} + \left( {P_{3} \sigma_{s}^{2} } \right)\mu_{2y}^{\prime } \left\{ {P\left( {\alpha^{2} - \beta^{2} } \right) + \beta^{2} } \right\}} \right]}}{{\alpha \beta \left( {\alpha + \beta } \right)\sigma_{s}^{2} }}$$

Using (101) and (102), the mathematical expression for *ϕ* can be written as:

103 $$\phi_{{\left( {NS/M_{4} } \right)}} = \frac{1}{{\left( {w_{1} + w_{2} } \right)}}\left( {w_{1} E_{{\left( {NS/M_{4} } \right)}} + w_{2} P_{{\left( {NS/M_{4} } \right)}} } \right)$$

#### The $\phi$ Measure of Gupta et al. ^16^ Model relative to the ***M***_***4***_

The efficiency and privacy of the Gupta et al. ^16^ model relative to the *M*_*4*_ using (13), (14), (21) and (22) can be expressed as:

104 $$E_{{\left( {G/M_{4} } \right)}} = \frac{{\left[ {\sigma_{y}^{2} + W\sigma_{s}^{2} + W\left( {1 - A} \right)\sigma_{t}^{2} \mu_{2y}^{\prime } } \right]}}{{\sigma_{y}^{2} + P_{2} \mu_{2y}^{\prime } \sigma_{t}^{2} + \left( {P_{3} \sigma_{s}^{2} } \right)\mu_{2y}^{\prime } \left\{ {P\left( {\alpha^{2} - \beta^{2} } \right) + \beta^{2} } \right\}}}$$

and

105 $$P_{{\left( {G/M_{4} } \right)}} = \frac{{P_{2} \mu_{2y}^{\prime } \sigma_{t}^{2} + \left( {P_{3} \sigma_{s}^{2} } \right)\mu_{2y}^{\prime } \left\{ {P\left( {\alpha^{2} - \beta^{2} } \right) + \beta^{2} } \right\}}}{{\left[ {\sigma_{s}^{2} + \left( {1 - A} \right)\sigma_{t}^{2} \mu_{2y}^{\prime } } \right]}}$$

Using (104) and (105), the mathematical expression for *ϕ* can be written as:

106 $$\phi_{{\left( {G/M_{4} } \right)}} = \frac{1}{{\left( {w_{1} + w_{2} } \right)}}\left( {w_{1} E_{{\left( {G/M_{4} } \right)}} + w_{2} P_{{\left( {G/M_{4} } \right)}} } \right)$$

**Fig. 4:**
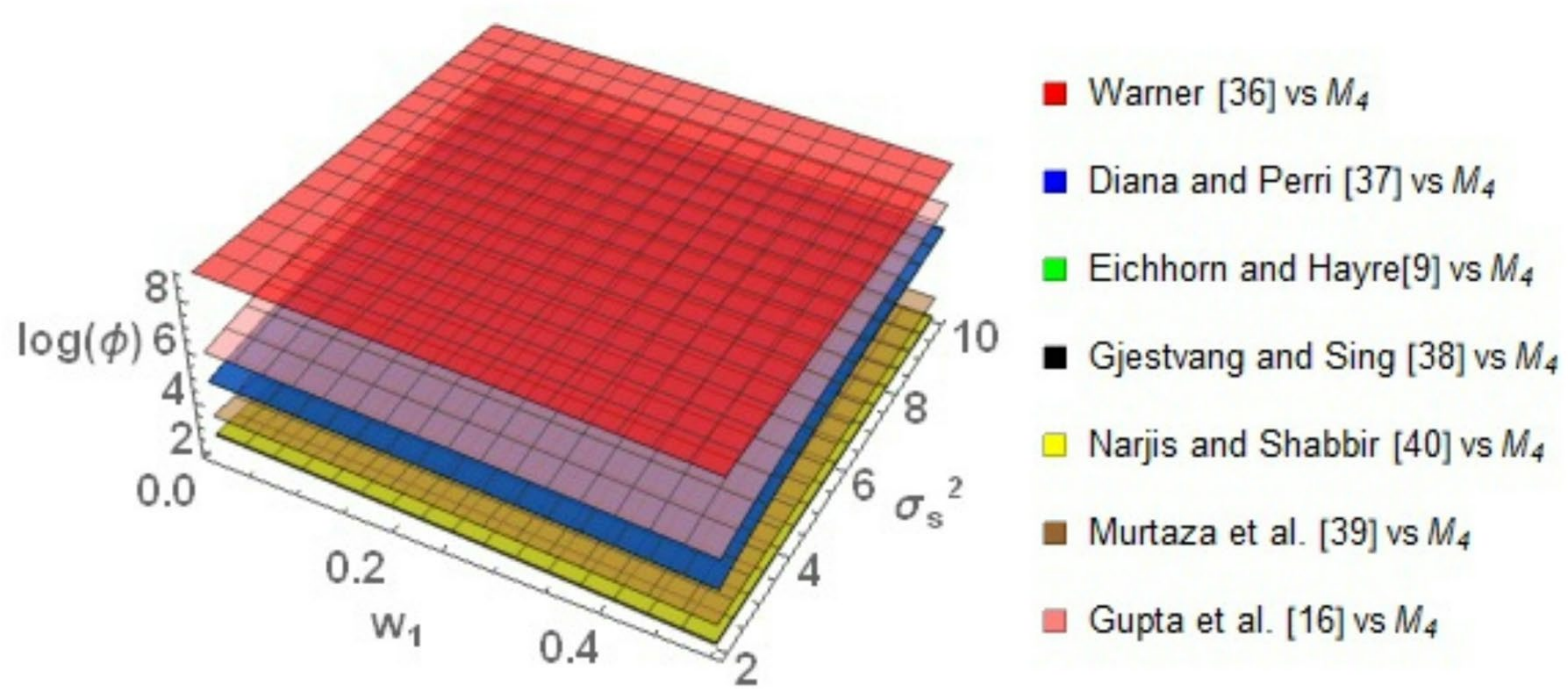
Behavior of $\log \left( \phi \right)$ for Model *M*_*4*_ relative to the existing models for $\sigma_{y}^{2} = 2$, $\mu_{y} = 5$, $\sigma_{T}^{2} = 25$, $\alpha = 25$, $\beta = 25$, $\gamma = 5$, $P = 0.4$, $P_{1} = 0.4$, $P_{2} = 0.6$, $A = 0.7$ A = 0.7, and $W = 0.2.$.

Figure 4 compares the $\log \left( \phi \right)$ behavior of the proposed model *M*_*4*_ with the existing models. The graphical analysis obviously demonstrates that *M*_*4*_ always performs better than the comparing models, as reflected by the positive values of $\log \left( \phi \right)$. Compared to Warner ^35^, *M*_*4*_ exhibits superior performance at smaller values of the weighting parameter $w_{1}$, with $\log \left( \phi \right)$ up to about 8.5. Even as $w_{1}$ varies from 0.2 to 0.8, the value of $\log \left( \phi \right)$ is larger , about 8.5, illustrating that *M*_*4*_ still remains efficient over the entire range of values of $w_{1}$ and if $\sigma_{S}^{2}$ increases, the $\log \left( \phi \right)$ remains constant and greater than zero.

As compared to Eichhorn and Hayre ^9^, Diana and Perri ^36^ and Gupta et al. ^16^, *M*_*4*_ consistently performs better over the entire ranges of $w_{1}$ and $\sigma_{S}^{2}$. This suggests superb stability and robustness in performance. It is also noteworthy to see that the value of $\log \left( \phi \right)$ increases with an increase in $\sigma_{S}^{2}$ and it decreases with an increase in the values of $w_{1}$. This suggests that *M*_*4*_ works very well. Comparison with Gjestvang and Singh ^37^, Narjis and Shabbir ^39^, and Murtaza et al. ^38^ reveals that *M*_*4*_ has excellent and consistent performance with low values of $w_{1}$. Particularly, the model results in positive values of $\log \left( \phi \right)$ for $w_{1}$ 0.2 to 0.8, and $\sigma_{S}^{2}$ 2 to 20. As can be seen in Fig. 4, although $\log \left( \phi \right)$ falls slightly with the increase of $w_{1}$, it remains positive, demonstrating the consistent performance of *M*_*4*_.

### Comparison of proposed models with each other

Now, we present the pairwise comparison of the proposed models in the following sub-sub-sections.

#### The $\phi$ measure of ***M***_***2***_ relative to the ***M***_***1***_

The efficiency and privacy of the *M*_*2*_ model relative to the *M*_*1*_ using (15), (16), (17) and (18) can be expressed as:

107 $$E_{{\left( {M_{2} /M_{1} } \right)}} = \frac{{\left[ {\sigma_{y}^{2} + P_{2} \mu_{2y}^{\prime } \left\{ {P\sigma_{t}^{2} + \left( {1 - P} \right)\sigma_{s}^{2} } \right\}} \right]}}{{\left[ {\sigma_{y}^{2} + \mu_{2y}^{\prime } \left( {P_{2} \sigma_{s}^{2} } \right)\left\{ {P\left( {\alpha^{2} - \beta^{2} } \right) + \beta^{2} } \right\}} \right]}}$$

_and_

108 $$P_{{\left( {M_{2} /M_{1} } \right)}} = \frac{{\left[ {\left( {P_{2} \sigma_{s}^{2} } \right)\left\{ {P\left( {\alpha^{2} - \beta^{2} } \right) + \beta^{2} } \right\}} \right]}}{{\left[ {P_{2} \left\{ {P\sigma_{t}^{2} + \left( {1 - P} \right)\sigma_{s}^{2} } \right\}} \right]}}$$

Using (107) and (108), the mathematical expression for *ϕ* can be written a

109 $$\phi_{{\left( {M_{2} /M_{1} } \right)}} = \frac{1}{{\left( {w_{1} + w_{2} } \right)}}\left( {w_{1} E_{{\left( {M_{2} /M_{1} } \right)}} + w_{2} P_{{\left( {M_{2} /M_{1} } \right)}} } \right)$$

#### The $\phi$ measure of ***M***_***3***_ relative to the ***M***_***1***_

The efficiency and privacy of the *M*_*3*_ model relative to the M_1_ using the Eq. (15), (16), (19) and (20) can be expressed as:

110 $$E_{{\left( {M_{3} /M_{1} } \right)}} = \frac{{\left[ {\sigma_{y}^{2} + P_{2} \sigma_{s}^{2} + P_{3} \mu_{2y}^{\prime } \left\{ {P\sigma_{t}^{2} + \left( {1 - P} \right)\sigma_{s}^{2} } \right\}} \right]}}{{\left[ {\sigma_{y}^{2} + \mu_{2y}^{\prime } \left( {P_{2} \sigma_{s}^{2} } \right)\left\{ {P\left( {\alpha^{2} - \beta^{2} } \right) + \beta^{2} } \right\}} \right]}}$$

and

111 $$P_{{\left( {M_{3} /M_{1} } \right)}} = \frac{{\left[ {\mu_{2y}^{\prime } \left( {P_{2} \sigma_{s}^{2} } \right)\left\{ {P\left( {\alpha^{2} - \beta^{2} } \right) + \beta^{2} } \right\}} \right]}}{{\left[ {P_{2} \sigma_{s}^{2} + P_{3} \mu_{2y}^{\prime } \left\{ {P\sigma_{t}^{2} + \left( {1 - P} \right)\sigma_{s}^{2} } \right\}} \right]}}$$

Using (110) and (111), the mathematical expression for *ϕ* can be written as:

112 $$\phi_{{\left( {Ml/M_{1} } \right)}} = \frac{1}{{\left( {w_{1} + w_{2} } \right)}}\left( {w_{1} E_{{\left( {M_{3} /M_{1} } \right)}} + w_{2} P_{{\left( {M_{3} /M_{1} } \right)}} } \right)$$

#### The $\phi$ measure of ***M***_***4***_ relative to the ***M***_***1***_

The efficiency and privacy of the *M*_*4*_ model relative to the *M*_*1*_ using (15), (16), (21) and (22) can be expressed as:

113 $$E_{{\left( {M_{4} /M_{1} } \right)}} = \frac{{\left[ {\sigma_{y}^{2} + P_{2} \mu_{2y}^{\prime } \sigma_{t}^{2} + \left( {P_{3} \sigma_{s}^{2} } \right)\mu_{2y}^{\prime } \left\{ {P\left( {\alpha^{2} - \beta^{2} } \right) + \beta^{2} } \right\}} \right]}}{{\left[ {\sigma_{y}^{2} + \mu_{2y}^{\prime } \left( {P_{2} \sigma_{s}^{2} } \right)\left\{ {P\left( {\alpha^{2} - \beta^{2} } \right) + \beta^{2} } \right\}} \right]}}$$

and

114 $$P_{{\left( {M_{4} /M_{1} } \right)}} = \frac{{\left[ {\left( {P_{2} \sigma_{s}^{2} } \right)} \right]\left\{ {P\left( {\alpha^{2} - \beta^{2} } \right) + \beta^{2} } \right\}}}{{\left[ {P_{2} \sigma_{t}^{2} + \left( {P_{3} \sigma_{s}^{2} } \right)\left\{ {P\left( {\alpha^{2} - \beta^{2} } \right) + \beta^{2} } \right\}} \right]}}$$

Using (113) and (114), the mathematical expression for *ϕ* can be written as:

115 $$\phi_{{\left( {M_{4} /M_{1} } \right)}} = \frac{1}{{\left( {w_{1} + w_{2} } \right)}}\left( {w_{1} E_{{\left( {M_{4} /M_{1} } \right)}} + w_{2} P_{{\left( {M_{4} /M_{1} } \right)}} } \right)$$

(Fig. 5, 6, 7).

**Fig. 5:**
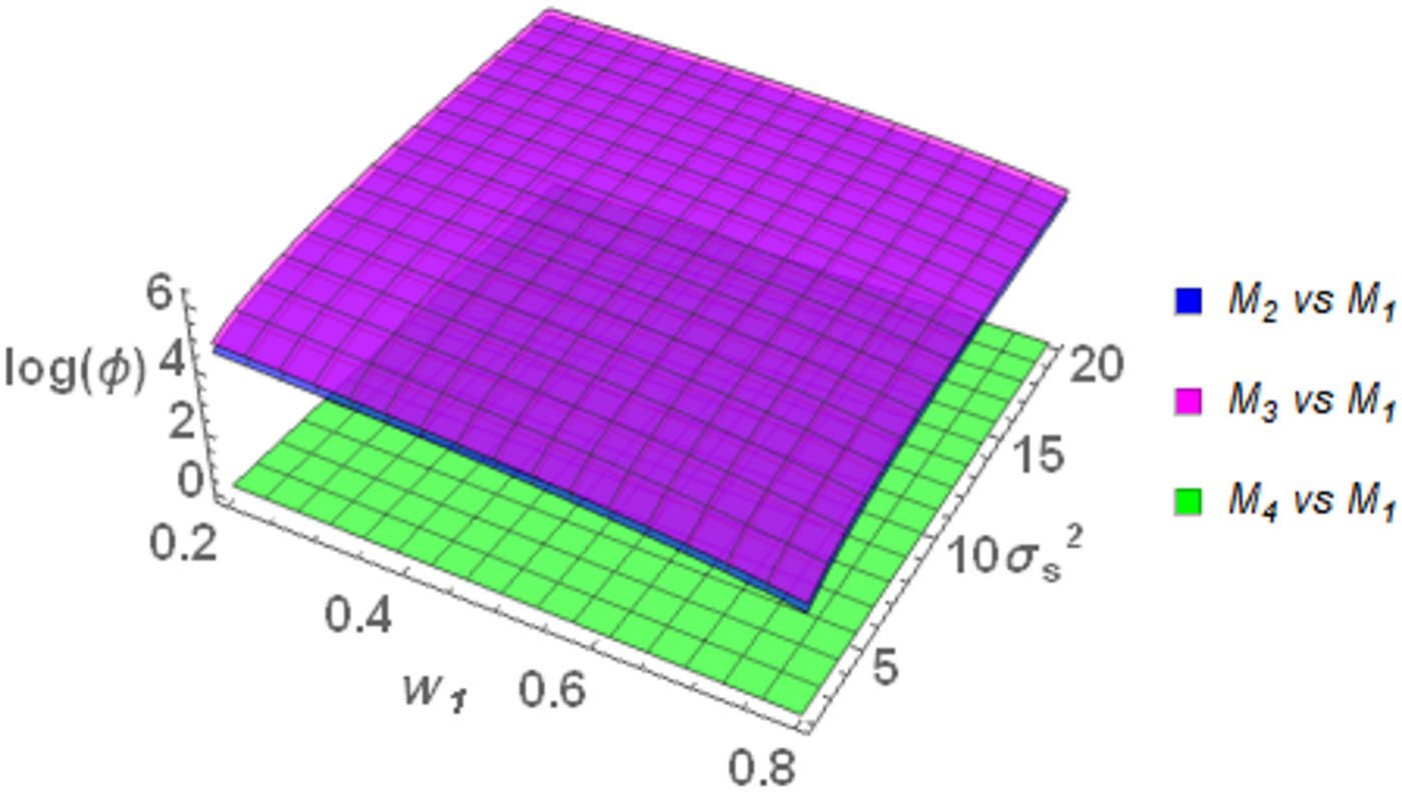
Behavior of $\log \left( \phi \right)$ for Model *M*_*1*_ relative to *M*_*2*_ , *M*_*3*_ and *M*_*4*_ for $\sigma_{y}^{2} = 2$, $\mu_{y} = 5$, $\sigma_{T}^{2} = 25$, $\alpha = 25$, $\beta = 25$, $\gamma = 5$, $P = 0.4$, $P_{1} = 0.4$, $P_{2} = 0.6$, $A = 0.7$ A = 0.7, and $W = 0.2.$.

When comparing *M*_1_ with *M*_*2*_ and* M*_*3*_ to determine its internal consistency and performance for different parameter values,* M*_*1*_ is found to be performing relatively better, especially with lower values of weight parameter $w_{1}$. At $w_{1} = 0.2$, $\log \left( \phi \right)$ goes up to around 4, and even when $w_{1}$ rises to 0.8, $\log \left( \phi \right)$ is still positive. This proves that *M*_*1*_ can keep high efficiency and privacy over the whole ranges of $w_{1}$. In addition, as the variance parameter $\sigma_{S}^{2}$ increases from 2 to 20, $\log \left( \phi \right)$ also increases, supporting the finding that *M*_*1*_ʹ performs relatively better than *M*_*2*_ and* M*_*3.*_

Then, when we compare *M*_4_ with *M*_*1*_, we can see that $\log \left( \phi \right)$ is positive when $w_{1}$ is less than 0.5 which shows that *M*_*1*_ performs better than *M*_*4*_. On the other hand, if $w_{1}$ is greater than 0.5 then $\log \left( \phi \right)$ remains negative which indicate that *M*_*4*_ performs well as compared to *M*_*1.*_

#### The $\phi$ measure of ***M***_***2***_ relative to the ***M***_***3***_

The efficiency and privacy of the *M*_*2*_ relative to the *M*_*3*_ using the (17), (18), (19) and (20) can be expressed as:

116 $$E_{{\left( {M_{2} /M_{3} } \right)}} = \frac{{\left[ {\sigma_{y}^{2} + \mu_{2y}^{\prime } P_{2} \left\{ {P\sigma_{t}^{2} + \left( {1 - P} \right)\sigma_{s}^{2} } \right\}} \right]}}{{\left[ {\sigma_{y}^{2} + P_{2} \sigma_{s}^{2} + P_{3} \mu_{2y}^{\prime } \left\{ {P\sigma_{t}^{2} + \left( {1 - P} \right)\sigma_{s}^{2} } \right\}} \right]}}$$

and

117 $$P_{{\left( {M_{2} /M_{3} } \right)}} = \frac{{P_{2} \sigma_{s}^{2} + P_{3} \mu_{2y}^{\prime } \left\{ {P\sigma_{t}^{2} + \left( {1 - P} \right)\sigma_{s}^{2} } \right\}}}{{\mu_{2y}^{\prime } P_{2} \left\{ {P\sigma_{t}^{2} + \left( {1 - P} \right)\sigma_{s}^{2} } \right\}}}$$

Using (116) and (117), the mathematical expression for *ϕ* can be written as:

118 $$\phi_{{\left( {M_{2} /M_{3} } \right)}} = \frac{1}{{\left( {w_{1} + w_{2} } \right)}}\left( {w_{1} E_{{\left( {M_{2} /M_{3} } \right)}} + w_{2} P_{{\left( {M_{2} /M_{3} } \right)}} } \right)$$

**Fig. 6:**
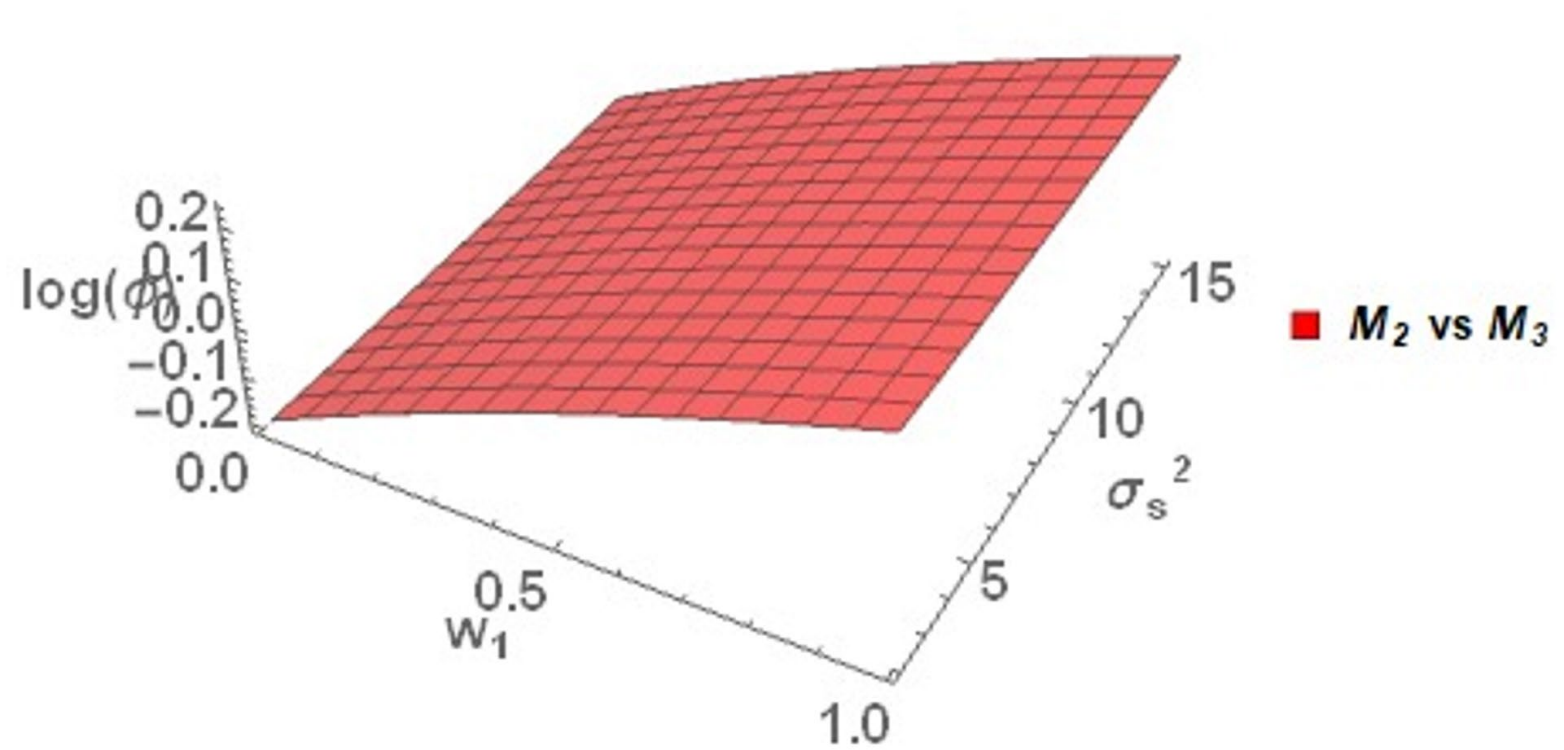
Behavior of $\log \left( \phi \right)$ for Model *M*_***2***_ relative to *M*_***3***_ for $\sigma_{y}^{2} = 2$, $\mu_{y} = 5$, $\sigma_{T}^{2} = 25$, $\alpha = 25$, $\beta = 25$, $\gamma = 5$, $P = 0.4$, $P_{1} = 0.4$, $P_{2} = 0.6$, $A = 0.7$ A = 0.7, and $W = 0.2.$.

Two competing models *M*_2_ and *M*_3_, are compared using the $\log \left( \phi \right)$ criterion. A negative value of $\log \left( \phi \right)$ indicates that *M*_2_ performs relatively better, whereas a positive value favors *M*_3_. Furthermore, *M*_2_ is preferred when $w_{1} < 0.4$. In contrast, values of $w_{1} \ge 0.4$ support the superiority of *M*_3_, which is consistent with the positive $\log \left( \phi \right)$.

#### The $\phi$ measure of ***M***_***2***_ relative to the ***M***_***4***_

The efficiency and privacy of the *M*_*2*_ relative to the *M*_*4*_ using (17), (18), (21) and (22) can be expressed as:

119 $$E_{{\left( {M_{2} /M_{4} } \right)}} = \frac{{\left[ {\sigma_{y}^{2} + P_{2} \mu_{2y}^{\prime } \left\{ {P\sigma_{t}^{2} + \left( {1 - P} \right)\sigma_{s}^{2} } \right\}} \right]}}{{\left[ {\sigma_{y}^{2} + P_{2} \mu_{2y}^{\prime } \sigma_{t}^{2} + \left( {P_{3} \sigma_{s}^{2} } \right)\mu_{2y}^{\prime } \left\{ {P\left( {\alpha^{2} - \beta^{2} } \right) + \beta^{2} } \right\}} \right]}}$$

_and_

120 $$P_{{\left( {M_{2} /M_{4} } \right)}} = \frac{{\left[ {P_{2} \sigma_{t}^{2} + \left( {P_{3} \sigma_{s}^{2} } \right)\left\{ {P\left( {\alpha^{2} - \beta^{2} } \right) + \beta^{2} } \right\}} \right]}}{{\left[ {P_{2} \left\{ {P\sigma_{t}^{2} + \left( {1 - P} \right)\sigma_{s}^{2} } \right\}} \right]}}$$

Using (119) and (120), the mathematical expression for *ϕ* can be written as

121 $$\phi_{{\left( {M_{2} /M_{4} } \right)}} = \frac{1}{{\left( {w_{1} + w_{2} } \right)}}\left( {w_{1} E_{{\left( {M_{2} /M_{4} } \right)}} + w_{2} P_{{\left( {M_{2} /M_{4} } \right)}} } \right)$$

#### The $\phi$ measure of ***M***_***3***_ relative to the ***M***_***4***_

The efficiency and privacy of the *M*_*3*_ model relative to the *M*_*4*_ using (19), (20), (21) and (22) can be expressed as:

122 $$E_{{\left( {M_{3} /M_{4} } \right)}} = \frac{{\left[ {\sigma_{y}^{2} + P_{2} \sigma_{s}^{2} + P_{3} \mu_{2y}^{\prime } \left\{ {P\sigma_{t}^{2} + \left( {1 - P} \right)\sigma_{s}^{2} } \right\}} \right]}}{{\left[ {\sigma_{y}^{2} + P_{2} \mu_{2y}^{\prime } \sigma_{t}^{2} + \left( {P_{3} \sigma_{s}^{2} } \right)\mu_{2y}^{\prime } \left\{ {P\left( {\alpha^{2} - \beta^{2} } \right) + \beta^{2} } \right\}} \right]}}$$

and

123 $$P_{{\left( {M_{3} /M_{4} } \right)}} = \frac{{\left[ {P_{2} \mu_{2y}^{\prime } \sigma_{t}^{2} + \left( {P_{3} \sigma_{s}^{2} } \right)\mu_{2y}^{\prime } \left\{ {P\left( {\alpha^{2} - \beta^{2} } \right) + \beta^{2} } \right\}} \right]}}{{\left[ {P_{2} \sigma_{s}^{2} + P_{3} \mu_{2y}^{\prime} \left\{ {P\sigma_{t}^{2} + \left( {1 - P} \right)\sigma_{s}^{2} } \right\}} \right]}}$$

Using (122) and (123), the mathematical expression for *ϕ* can be written as:

124 $$\phi_{{\left( {M_{3} /M_{4} } \right)}} = \frac{1}{{\left( {w_{1} + w_{2} } \right)}}\left( {w_{1} E_{{\left( {M_{3} /M_{4} } \right)}} + w_{2} P_{{\left( {M_{3} /M_{4} } \right)}} } \right)$$

We start with the comparison of Model *M*_4_ with *M*_2_ and *M*_3_ to assess its internal consistency as well as performance for different values of parameters. Interestingly, Model *M*_4_ performs very well, especially at smaller values of the weighting parameter $w_{1}$. For example, when $w_{1} = 0.2$, $\log \left( \phi \right)$ attains the value of about 4, and even when $w_{1}$ is increased to 0.8, $\log \left( \phi \right)$ remains positive.

**Fig. 7:**
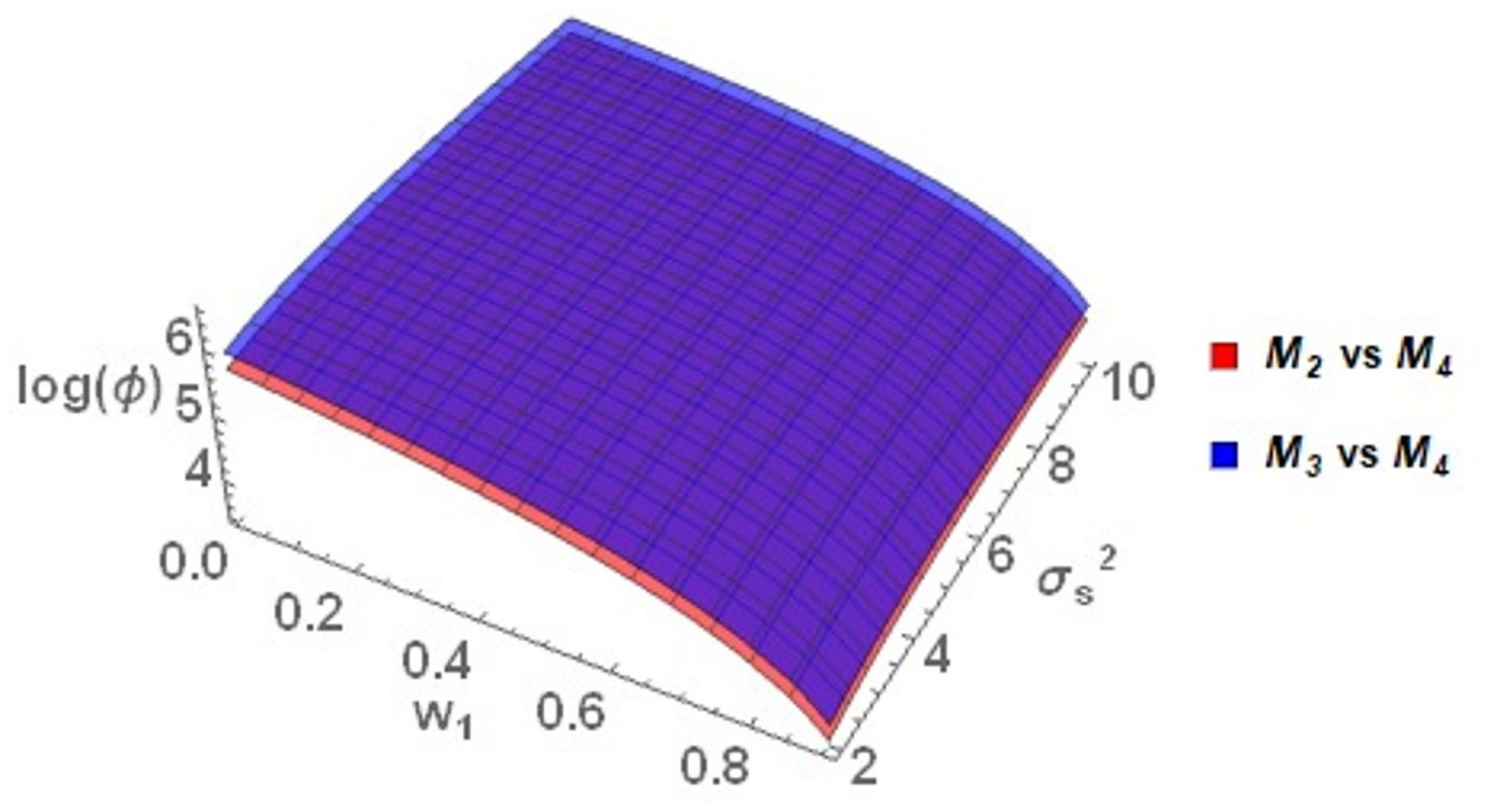
Behavior of $\log \left( \phi \right)$ for Model *M*_*2*_ and *M*_*3*_ relative to *M*_*4*_ for $\sigma_{y}^{2} = 2$, $\mu_{y} = 5$, $\sigma_{T}^{2} = 25$, $\alpha = 25$, $\beta = 25$, $\gamma = 5$, $P = 0.4$, $P_{1} = 0.4$, $P_{2} = 0.6$, $A = 0.7$ A = 0.7, and $W = 0.2.$.

## Practical guidelines for model selection

Guided by the comparative results in this study, we can draw the following guidelines for practitioners in selecting an appropriate scrambling model for a given sensitive survey application.

- When privacy is the primary concern, that is, $w_{1} < 0.5$, Model *M*_1_ is recommended due to its stable performance and relatively high efficiency across a wide range of variances of scrambling variable. Its robustness makes it particularly suitable for highly sensitive questions where strong privacy protection is required.
- Models *M*_2_ and *M*_3_ are suitable alternatives in privacy-focused settings when moderate efficiency gains are desired. Among these, *M*_2_ is preferable when the variance of the scrambling variable is low to moderate, as it exhibits slightly better efficiency under controlled randomization. *M*_3_ may be favored when greater robustness is required, as it shows less sensitivity to changes in the scrambling variance, albeit at a small cost in efficiency.
- When efficiency becomes more important than privacy, that is $w_{1} > 0.5$, Model *M*_4_ consistently outperforms competing models and is therefore recommended in applications where estimation precision is the dominant objective.

## Conclusion

In conclusion, from a rigorous comparative study based on six distinct graphical assessments, we find that the suggested models particularly Model *M*_*1*_ and Model *M*_*4*_ are more efficient and privacy-preserving. The utilization of the combined performance measure $\log \left( \phi \right)$, which evaluates relative efficiency and privacy preservation together, accentuates the robustness of these models for a range of values of weight parameters $w_{1}$ and design parameter $\sigma_{S}^{2}$

Model *M*_*1*_ is stable and efficient even under low weight conditions and higher variance of scrambling variable *S*, while Model *M*_*4*_ performs better than all alternatives if $w_{1} > 0.5$. Models *M*_*2*_ and *M*_*3*_ are also very encouraging, frequently performing better than traditional models. The empirical results presented in Figs. 1, 2, 3, 4, 5, 6, 7 demonstrate that each of the four proposed randomized response models substantially improves the trade-off between estimator accuracy and respondent confidentiality when compared with existing alternatives. As a result of their consistently high $\log \left( \phi \right)$ values and unbiased estimators, these models are exceedingly appropriate for survey applications with sensitive quantitative data. Consequently, these models may be considered an important contribution to the area of quantitative randomized response modeling. Overall, the choice of model should be guided by the intended balance between privacy and efficiency, with Models *M*_1_–*M*_3_ recommended for privacy-dominated surveys and Model *M*_4_ preferred when efficiency is the primary objective.

## Data Availability

Data is available on demand, email can be sent to [Talha.omer@lnu.se] (mailto:Talha.omer@lnu.se), for the data.
